# Semaphorin 3E–Plexin D1 Axis Drives Lung Fibrosis through ErbB2‐Mediated Fibroblast Activation

**DOI:** 10.1002/advs.202415007

**Published:** 2025-03-20

**Authors:** Zhesong Deng, Jinkun Chen, Ruonan Yang, Yuan Zhan, Shanshan Chen, Jiaheng Zhang, Hao Fu, Yiya Gu, Qian Huang, Jixing Wu, Lianyu Shan, Abdelilah Soussi Gounni, Jungang Xie

**Affiliations:** ^1^ Department of Respiratory and Critical Care Medicine National Clinical Research Center of Respiratory Disease Key Laboratory of Pulmonary Diseases of Health Ministry Tongji Hospital Tongji Medical College Huazhong University of Science and Technology Wuhan Hubei 430030 China; ^2^ Lawrence Bloomberg University of Toronto 155 College Street, Suite 130 Toronto ON M5T 1P8 Canada; ^3^ Department of Respiratory and Critical Care Medicine The First Affiliated Hospital of Chongqing Medical University Chongqing 401122 China; ^4^ Department of Immunology Max Rady College of Medicine Rady Faculty of Health Sciences University of Manitoba Winnipeg MB R3E 0T5 Canada

**Keywords:** idiopathic pulmonary fibrosis, myofibroblast differentiation, Plexin D1, Sema3E

## Abstract

Idiopathic pulmonary fibrosis (IPF) is characterized by excessive fibroblast recruitment and persistent extracellular matrix deposition at sites of tissue injury, leading to severe morbidity and mortality. However, the precise mechanisms by which fibroblasts contribute to IPF pathogenesis remain poorly understood. The study reveals that Sema3E and its receptor Plexin D1 are significantly overexpressed in the lungs of IPF patients and bleomycin (BLM)‐induced lung fibrotic mice. Elevated plasma levels of Sema3E in IPF patients are negatively correlated with lung function. Importantly, Sema3E in IPF lungs predominantly exists as the P61‐Sema3E. The knockdown of Sema3E or Plexin D1 effectively inhibits fibroblast activation, proliferation, and migration. Mechanistically, Furin‐mediated cleavage of P87‐Sema3E into P61‐Sema3E drives these pro‐fibrotic activities, with P61‐Sema3E‐PlexinD1 axis promoting fibroblast activation, proliferation, and migration by affecting the phosphorylation of ErbB2, which subsequently activates the ErbB2 pathways. Additionally, Furin inhibition reduces fibroblast activity by decreasing P61‐Sema3E production. In vivo, both whole‐lung Sema3E knockdown and fibroblast‐specific Sema3E knockout confer protection against BLM‐induced lung fibrosis. These findings underscore the crucial role of the P61‐Sema3E‐Plexin D1 axis in IPF pathogenesis and suggest that targeting this pathway may hold promise for the development of novel therapeutic strategies for IPF treatment.

## Graphical abstract

TGF‐β1 stimulation in fibroblasts leads to increased Furin expression, Furin‐mediated cleavage of P87‐Sema3E to P61‐Sema3E, which affects fibroblast function through the autocrine pathway, and the P61‐Sema3E‐PlexinD1 axis promotes fibroblast activation, proliferation and migration by affecting ErbB2 phosphorylation, which in turn activates the ErbB2 pathway.

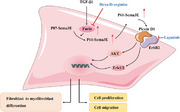

## 1 Introduction

Idiopathic pulmonary fibrosis (IPF) is a progressive and fatal interstitial lung disease with a high risk of mortality.^[^ ^1^ ^]^ However, the treatment options for patients with IPF are quite restricted. Pirfenidone and nintedanib are the only antifibrotic drugs available for clinical use in the treatment of IPF, yet their therapeutic effectiveness remains limited.^[^ ^2^ ^]^ IPF is characterized by epithelial cell injury and a significant number of fibroblasts undergoing differentiation into myofibroblasts.^[^ ^3^ ^]^ Activated fibroblasts, also referred to as myofibroblasts, demonstrate excessive migratory and proliferative capabilities, leading to their accumulation at the sites of tissue injury. Consequently, this triggers an exaggerated deposition of extracellular matrix (ECM), ultimately resulting in tissue structural destruction and fibrosis.^[^ ^4^ ^]^ Despite significant research efforts, the precise mechanisms underlying pulmonary fibrosis remain elusive.^[^ ^5^ ^]^ Therefore, it is imperative to investigate the various influencing factors associated with fibroblast differentiation, proliferation, and migration in IPF, as they hold the key to unraveling the intricate mechanisms underlying this fatal disease.

Semaphorins constitute a family of proteins that encompass both secreted and membrane‐bound signals that were initially linked to the regulation of neuronal development.^[^ ^6^ ^]^ Recent studies have revealed the widespread expression and diverse functions of the Semaphorin family, which exerts a broad spectrum of effects on pathophysiological processes, including tissue fibrosis, angiogenesis, tumor progression, and inflammation.^[^ ^7^, ^8^, ^9^, ^10^, ^11^, ^12^ ^]^ Semaphorin 4A and Semaphorin 7A are involved in the regulation of pulmonary fibrosis progression. In this study, we focused on Semaphorin 3E (Sema3E), another member of the Semaphorin family. Sema3E is a secreted protein that mediates its effects by binding to its receptor Plexin D1. Tumor‐related studies reported that Sema3E and its receptor Plexin D1 are significantly upregulated in various cancers such as colon cancer and metastatic breast cancer. They have been implicated in promoting tumor migration, angiogenesis, and epithelial–mesenchymal transition (EMT).^[^ ^13^, ^14^, ^15^ ^]^ Serum levels of Sema3E are markedly elevated in patients with Systemic Sclerosis (SSc) compared with those in control subjects.^[^ ^16^ ^]^ In studies pertaining to tissue fibrosis, Yagai et al.^[^ ^17^ ^]^ reported ameliorated liver fibrosis in Sema3E‐knockout mice compared to that in wild‐type mice in a chronic liver injury model, suggesting a potential association between Sema3E and tissue fibrosis. In contrast, in asthma, Sema3E administration inhibited airway remodeling and reduce collagen deposition.^[^ ^18^ ^]^ Sema3E function has exhibited conflicting results in studies of various fibrosis‐related diseases. This observation could be attributed to the presence of two distinct forms of Sema3E, each with different functional roles. Sema3E exists in its full‐length form, p87‐Sema3E, as well as a truncated proteolytic fragment, p61‐Sema3E, which is predominantly produced via Furin‐mediated cleavage. These forms likely exhibit differential biological activities, contributing to the diverse roles of Sema3E in various cellular processes.^[^ ^13^, ^19^, ^20^ ^]^ Both forms interact with the Plexin D1.^[^ ^13^, ^21^ ^]^ Previous studies have not investigated the function of different Sema3E forms in fibrosis‐related diseases, it is not clear which of these Sema3E forms is the main active form of pro‐fibrotic. The precise molecular mechanisms by which Sema3E influences pulmonary fibrosis have not yet been elucidated.

In this study, we investigated the differential expression of the Sema3E‐Plexin D1 axis in IPF and elucidated its role in promoting fibroblast differentiation, proliferation, and migration. Our preliminary findings from human lung tissue and plasma samples suggest that the elevated expression of Sema3E and its receptor Plexin D1 may be implicated in the pathogenesis of IPF, with Sema3E, particularly in its P61 form, emerging as the primary active form in promoting fibrosis. To substantiate this hypothesis, we established cell and mouse models, revealing that TGF‐β1 stimulation induces a significant increase in Furin expression in fibroblasts. This, in turn, upregulates P61‐Sema3E expression in fibroblasts, thereby promoting fibroblast differentiation, proliferation, and migration via autocrine signaling pathways, ultimately contributing to the pathogenesis of IPF. These findings enhance our understanding of IPF pathogenesis and present a potential target for therapeutic intervention in IPF management.

## 2 Results

### 2.1 Elevated Expression of Sema3E in Plasma and Lung Tissue from IPF Patients and Mice

**Figure 1:**
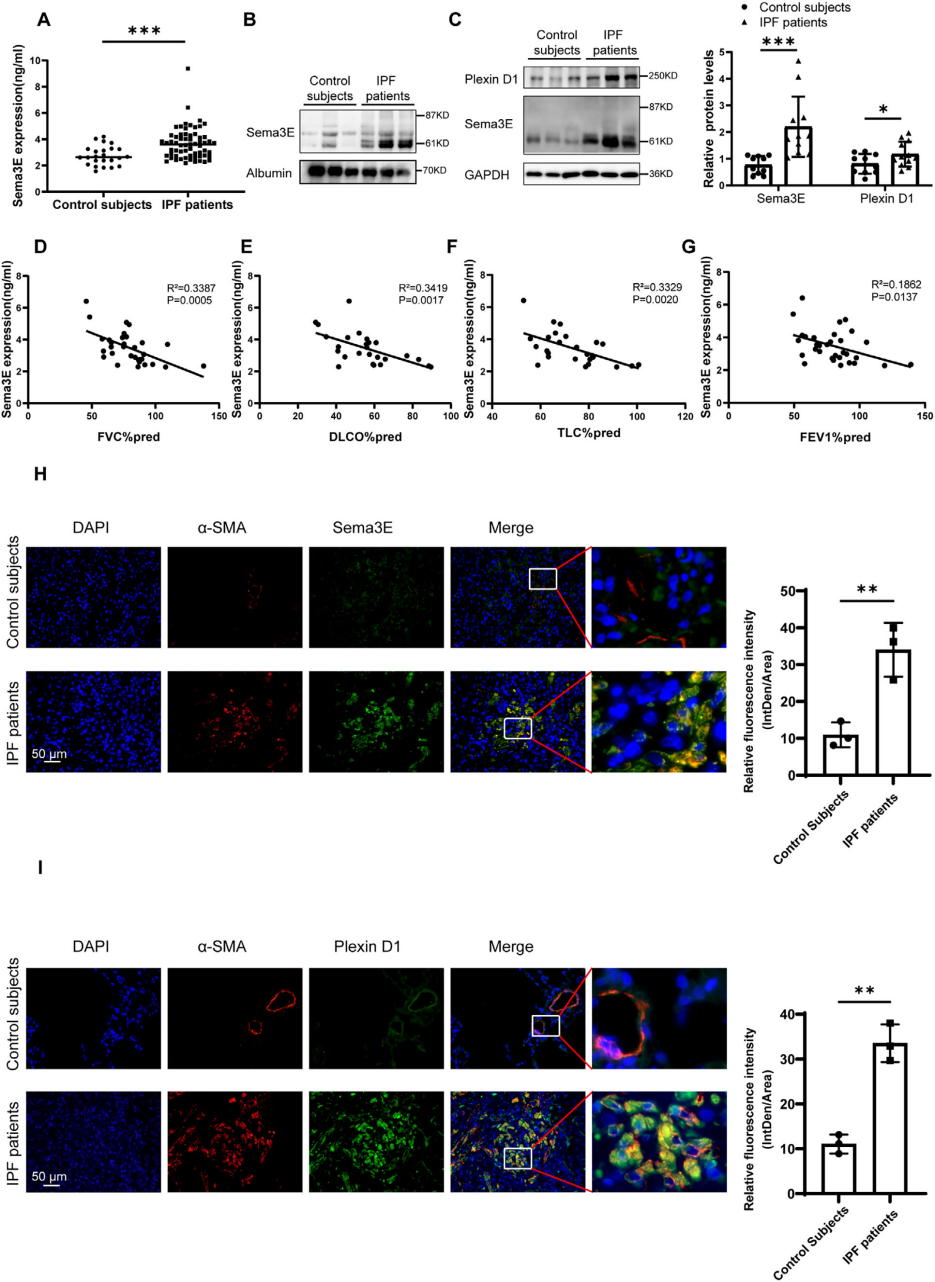
Analysis of Sema3E and Plexin D1 levels in IPF Patients. A) ELISA analysis comparing Sema3E levels in the plasma of patients with IPF (*n* = 63) and control subjects (*n* = 24). B) Representative western blot analysis depicting Sema3E expression in plasma samples from IPF patients. C) Representative western blot analysis illustrating Sema3E and Plexin D1 expression in lung tissue samples from control subjects (*n* = 10) and IPF patients (*n* = 12). D–G) Correlation analysis between Sema3E levels and clinical parameters including (D) FVC%pred, (E) DLCO%pred, (F) TLC%pred, and (G) FEV1%pred in IPF patients (*n* = 32). H) Co‐immunostaining of Sema3E and α‐SMA in lung sections from patients with IPF(*n* = 3) and control subjects(*n* = 3). I) Co‐immunostaining of Plexin D1 and α‐SMA in lung sections from patients with IPF(*n* = 3) and control subjects(*n* = 3). Nuclei were stained blue with Diamidino‐2‐phenylindole dihydrochloride (DAPI), and images were captured under original magnification ×400. Data are presented as the mean ± SEM. Statistical analyses were performed using unpaired t‐tests and Pearson's correlation. *p < 0.05, **p < 0.01, and ***p < 0.001 indicate significance levels. (FVC%pred: Forced Vital Capacity % predicted; TLC%pred: Total Lung Capacity % predicted; DLCO%pred: Diffusing Capacity of the Lung for Carbon Monoxide % predicted; FEV1%pred: Forced Expiratory Volume in 1 second % predicted.).

To investigate the expression of Sema3E and the receptor Plexin D1 in IPF patients, plasma samples were collected from 87 individuals, including 24 healthy controls and 63 IPF patients as detailed in Table S1 (Supporting Information). Age and gender were matched between the two groups, with IPF patients exhibiting lower FVC% pred and FEV1% pred than healthy controls. The lung tissue samples included 22 samples, with 10 from the control group and 12 from the IPF patients’ group as shown in Table S2 (Supporting Information). Subsequently, the expression of Sema3E in plasma and lung samples of IPF patients was examined using ELISA, immunofluorescent staining, and western blot. Our analysis revealed a significant increase in Sema3E expression in the plasma of IPF patients compared to healthy controls (**Figure** **1**A), with a negative correlation observed between Sema3E expression and TLC% pred, DLCO% pred, FEV1% pred, and FVC% pred in IPF patients (Figure 1D–G). Interestingly, based on the western blot results, it was observed that the predominant form of Sema3E in the plasma of IPF patients is P61‐Sema3E (Figure 1B). Further analysis of Sema3E and its receptor PlexinD1 in lung tissues of IPF patients revealed higher expression levels of P61‐Sema3E and Plexin‐D1 compared to healthy controls, consistent with the plasma findings (Figure 1C). The expression of the P87‐Sema3E was significantly lower in lung tissues of IPF patients compared to the P61‐Sema3E. These results indicate that the P61‐Sema3E is the predominant form in both lung tissues and plasma of patients with IPF. Furthermore, immunofluorescence co‐staining of lung sections showed that patients with IPF had higher levels of Sema3E and Plexin D1 compared to the control group, particularly in myofibroblasts (Figure 1H,I). In addition, we used CD68 to identify macrophages and E‐cadherin to identify alveolar epithelial cells. Immunofluorescence co‐staining of Sema3E with these markers revealed minimal Sema3E expression in macrophages and high expression in alveolar epithelial cells. However, in the fibrotic context, neither cell type exhibited significant changes in Sema3E levels (Figure S1A,B, Supporting Information).

**Figure 2:**
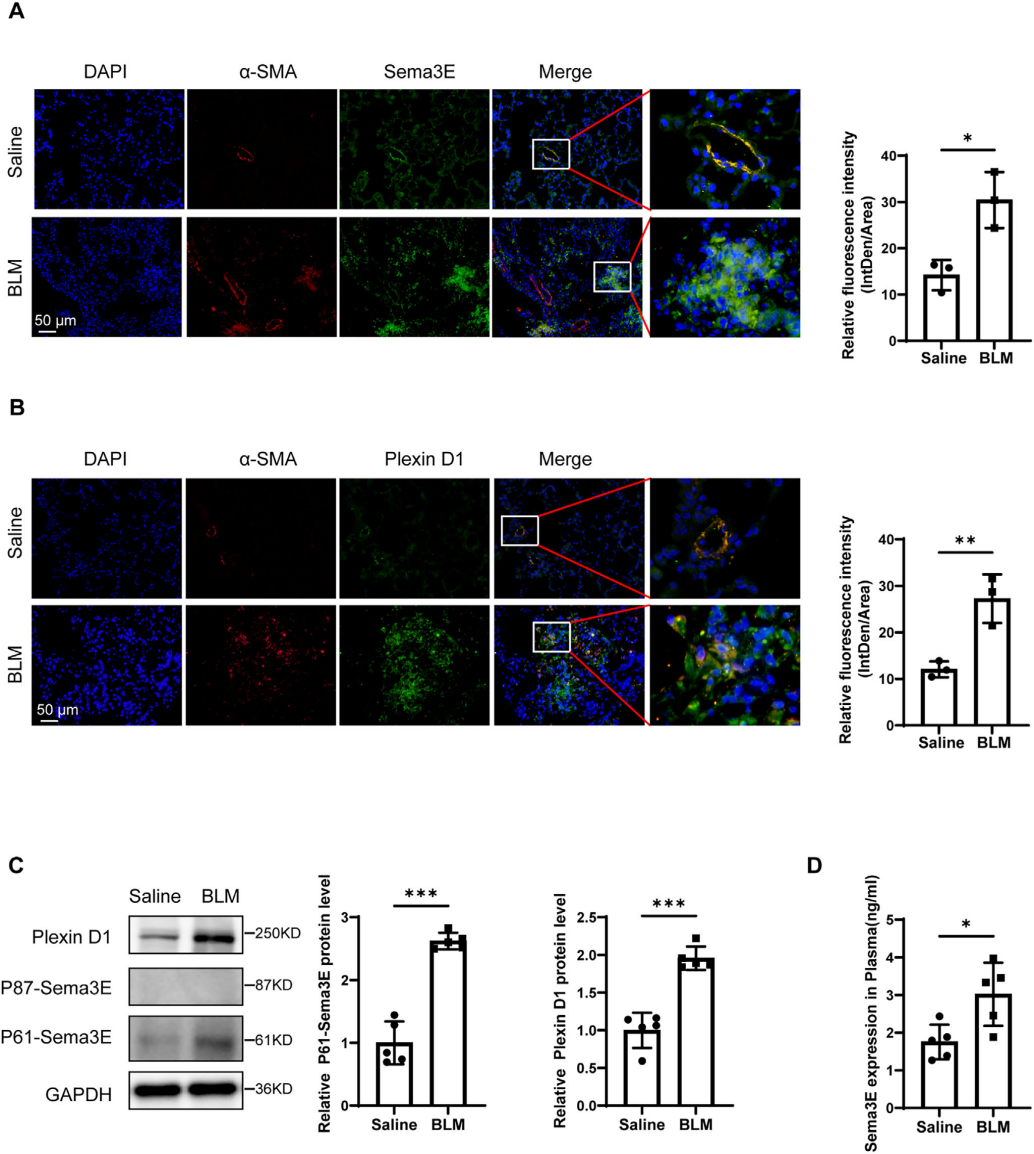
Analysis of Sema3E and Plexin D1 levels in mice with BLM induction. A) Co‐immunostaining of Sema3E and α‐SMA in lung sections from the BLM‐induced mouse model (*n* = 3) and saline controls (*n* = 3). B) Co‐immunostaining of Plexin D1 (*n* = 3) and α‐SMA (*n* = 3) in lung sections from the BLM‐induced mouse model and saline controls. Nuclei were counterstained blue with DAPI, and images were captured at an original magnification of ×400. C) Western blot analysis depicting the expression levels of Sema3E and Plexin D1 in lung homogenates from saline‐treated (*n* = 5) and BLM‐induced (*n* = 5) mouse models. D) ELISA analysis comparing Sema3E levels in the plasma of the saline‐treated mice (*n* = 5) and BLM‐induced mouse model(*n* = 5). Data are presented as the mean ± SEM. Statistical analyses were performed using unpaired t‐tests. *p < 0.05; **p < 0.01; ***p < 0.001.

To further elucidate the expression patterns of Sema3E and Plexin D1 in IPF, we conducted a comprehensive analysis using the BLM)‐induced mouse model. Employing both western blot and immunofluorescence, we assessed the protein expression of Sema3E and Plexin D1 in this mouse model. Consistent with the findings in human lung tissues, our results revealed robust protein expression of both Sema3E and PlexinD1 in the BLM‐induced mouse model (**Figure** **2**C). Notably, Sema3E predominantly manifested as the P61‐Sema3E. Furthermore, our immunofluorescence co‐staining demonstrated a distinct co‐localization of PlexinD1 and Sema3E with α‐SMA (Figure 2A,B), indicative of their association with myofibroblasts.

To further determine whether Sema3E is elevated during fibrotic progression, we measured its levels in the plasma of BLM‐induced fibrotic mice and control mice using ELISA. Plasma Sema3E levels were significantly elevated in fibrotic mice compared to controls, consistent with our previous findings in lung tissue and plasma from IPF patients (Figure 2D). Collectively, these findings underscore the relevance of Sema3E in fibrogenesis and strengthen its candidacy as a biomarker for pulmonary fibrosis.

### 2.2 Up‐Regulation of Sema3E is Induced by TGF‐β1 in Fibroblasts

**Figure 3:**
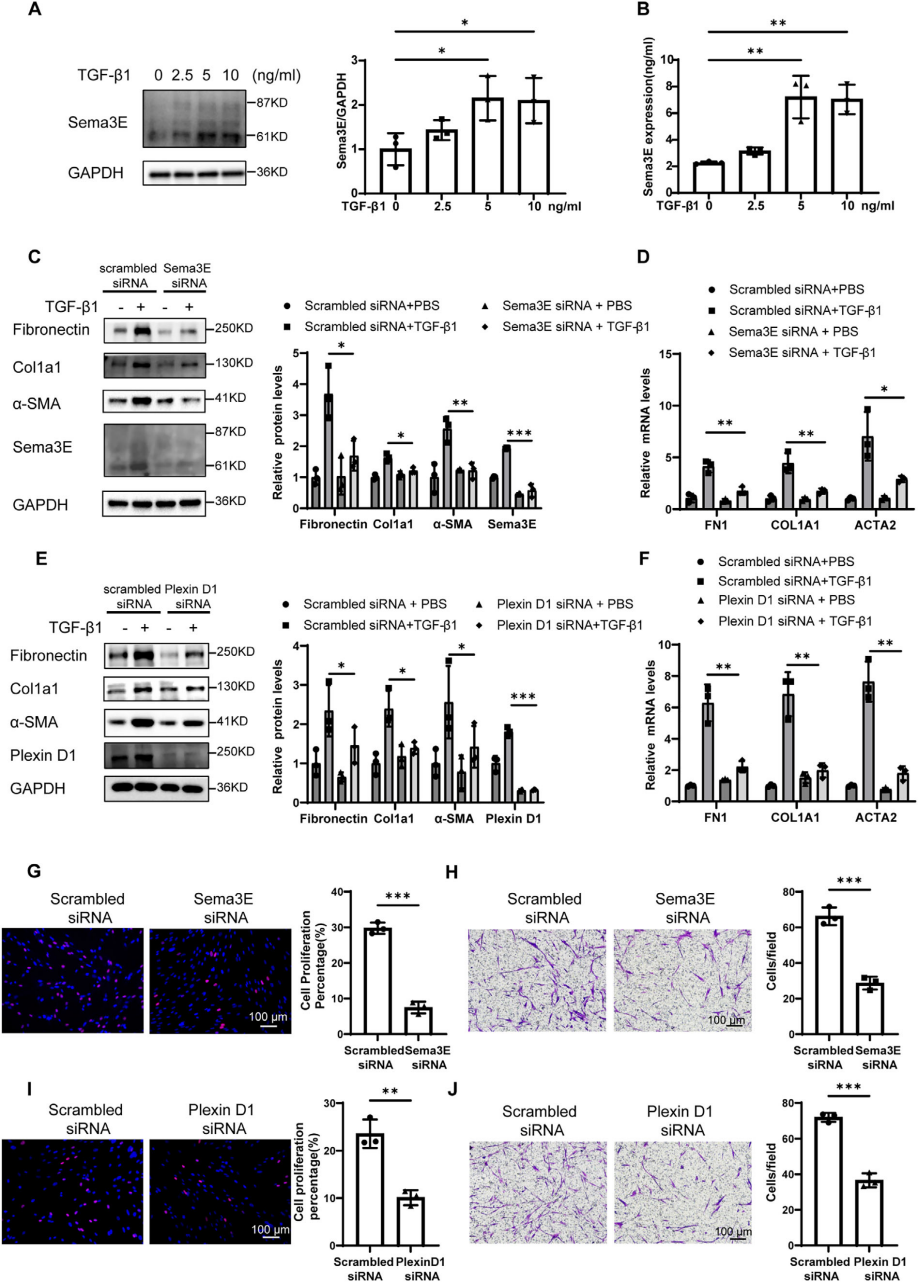
The impact of Sema3E‐Plexin D1 on the differentiation, proliferation, and migration of fibroblasts. A) Western blot analysis of Sema3E expression in primary human lung fibroblasts (PHLFs) following stimulation with different concentrations of TGFβ1 for 48 h. B) Measurement of Sema3E concentration in culture media by ELISA. PHLFs were stimulated with TGF‐β1, and Sema3E concentration was assessed after 48 h. C,D) Western blot and RT‐qPCR analysis of Fibronectin, Col1a1, and α‐SMA protein and mRNA expression in Sema3E siRNA or Scrambled siRNA‐treated PHLFs following TGF‐β1 induction. E,F) Western blot and RT‐qPCR analysis of Fibronectin, Col1a1, and α‐SMA protein and mRNA expression in PlexinD1 siRNA or Scrambled siRNA‐treated PHLFs following TGF‐β1 induction. G) Representative results for EdU staining in Sema3E siRNA or Scrambled siRNA‐treated PHLFs. H) Representative results for Transwell assay in Sema3E siRNA or Scrambled siRNA‐treated PHLFs. I) Representative results for EdU staining in Plexin D1 siRNA or Scrambled siRNA‐treated PHLFs. J) Representative results for Transwell assay in Plexin D1 siRNA or Scrambled siRNA‐treated PHLFs. Images were captured under original magnification ×200. Data are presented as the mean ± SEM of three independent experiments. Statistical analyses were performed using unpaired t‐tests and one‐way ANOVA test. *p < 0.05; **p < 0.01; ***p < 0.001.

As Sema3E is predominantly expressed in fibroblasts, we isolated and cultured primary fibroblasts from the lung tissue of patients with IPF. To induce fibroblast‐to‐myofibroblast differentiation, we stimulated the cells with TGF‐β1. Intracellular Sema3E expression in myofibroblasts was assessed using western blot analysis, while secreted Sema3E levels in cell supernatants were quantified by ELISA. Our results revealed a significant upregulation of both intracellular Sema3E expression in myofibroblasts and secreted Sema3E levels in cell supernatants following stimulation with TGF‐β1 (**Figure** **3**A,B). These findings underscore a significant upregulation of Sema3E expression following TGF‐β1‐induced fibroblast differentiation into myofibroblasts, indicating its potential role in the activation and function of fibroblasts in the context of IPF.

### 2.3 Sema3E is Involved in Fibroblast Proliferation, Migration, and Activation through Autocrine Signaling

According to previous studies, Sema3E knockout mice showed improved liver fibrosis compared to wild‐type mice in a chronic liver injury model.^[^ ^17^ ^]^ However, the function of the Sema3E‐Plexin D1 signaling pathway in pulmonary fibrosis remains unclear. To elucidate the role of Sema3E in fibroblast differentiation, we used TGF‐β1 stimulation of fibroblasts to induce fibroblast differentiation and employed siRNA targeting Sema3E to investigate its impact on lung fibrosis. Silencing Sema3E expression in primary human lung fibroblasts markedly reduced the differentiation of fibroblasts into myofibroblasts, as demonstrated by the lower protein levels of myofibroblast markers, including Fibronectin, Col1a1, and α‐SMA, RT‐qPCR analysis of Fn1, Col1a1, and Acta2 provided additional confirmation of this finding (Figure 3C,D). Collectively, these findings indicate that Sema3E deficiency impedes fibroblast differentiation, suggesting a pivotal role for Sema3E in this process.

Given that Plexin D1 serves as the receptor for Sema3E, we further examined the potential anti‐fibrotic effects of Plexin D1 silencing on myofibroblast activation. Utilizing siRNA targeting Plexin D1, we transfected primary human lung fibroblasts to assess its effects on pulmonary fibrosis. Remarkably, knockdown of Plexin D1 resulted in a reduction of the upregulated protein and mRNA expression of Fibronectin, Col1a1, and α‐SMA induced by TGF‐β1 (Figure 3E,F).

In addition, we evaluated the influence of Sema3E and Plexin D1 on the proliferation of primary fibroblasts derived from patients with IPF using EdU staining without TGF‐β1 stimulation. Remarkably, silencing of either Sema3E or Plexin D1 expression resulted in a notable reduction in the proliferation of primary human lung fibroblasts (Figure 3G,I). And we investigated the migratory capacity of primary human lung fibroblasts using Transwell assays without TGF‐β1 stimulation. The knockdown of Sema3E significantly attenuated the migration of primary fibroblasts from patients with IPF (Figure 3H). Similarly, the suppression of Plexin D1 expression resulted in a notable reduction in the migration of primary human lung fibroblasts (Figure 3J).

These collective findings strongly indicate that the Sema3E‐PlexinD1 signaling axis plays a crucial role in regulating fibroblast activation, migration, and proliferation, thereby implicating its potential involvement in the pathogenesis of pulmonary fibrosis.

### 2.4 P61‐Sema3E is the Main Pro‐Fibrotic Active Form of Sema3E

**Figure 4:**
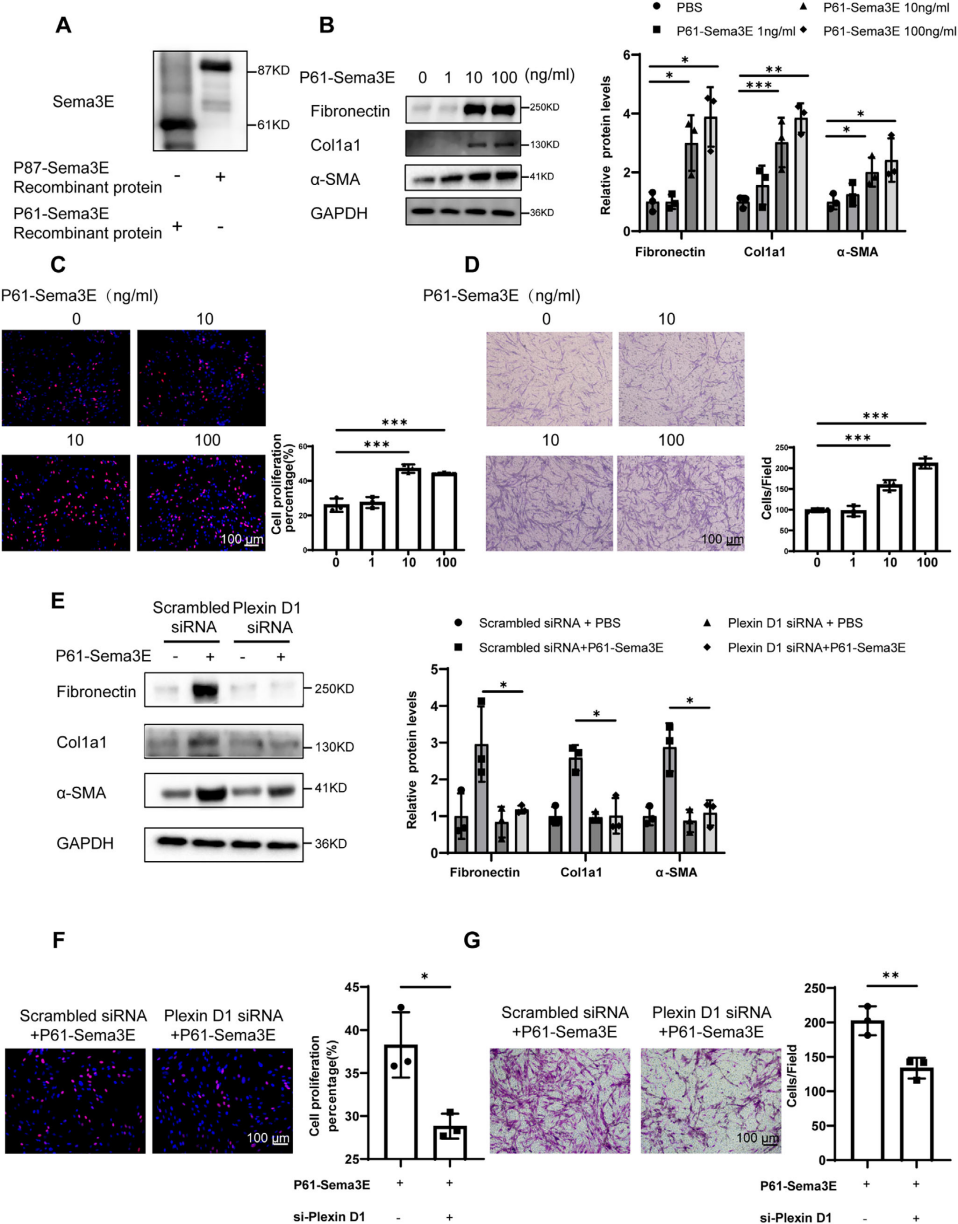
P61‐Sema3E promotes differentiation, proliferation, and migration of fibroblasts through Plexin D1. A) Verification of two recombinant proteins, P87‐Sema3E and P61‐Sema3E, by western blot. B) Western blot analysis of the levels of Fibronectin, Col1a1, and α‐SMA in primary human lung fibroblasts (PHLFs) after stimulation with different concentrations of P61‐Sema3E for 48 h. C) Representative results for EdU staining in PHLFs treated with different concentrations of P61‐Sema3E for 48 h. D) Representative results for Transwell assay in PHLFs treated with different concentrations of P61‐Sema3E for 48 h. E) Western blot analysis of Fibronectin, Col1a1, and α‐SMA expression in PlexinD1 siRNA or Scrambled siRNA‐treated PHLFs following P61‐Sema3E induction. F) Representative results for EdU staining in PlexinD1 siRNA or Scrambled siRNA‐treated PHLFs following P61‐Sema3E stimulation for 48 h. G) Representative results for Transwell assay in Plexin D1 siRNA or Scrambled siRNA‐treated PHLFs following P61‐Sema3E stimulation for 48 h. Images were captured under original magnification ×200. Data are presented as the mean ± SEM of three independent experiments. Statistical analyses were performed using unpaired t‐tests and one‐way ANOVA test. *p < 0.05; **p < 0.01; ***p < 0.001.

Given the significant increase in Sema3E levels observed in IPF patients, particularly in the form of the P61‐Sema3E, and considering previous reports indicating that P61‐Sema3E is the active form of Sema3E in cancer cells, our investigation aimed to delineate the functional significance of different forms of Sema3E in the pathogenesis of IPF. Based on our observation that P61‐Sema3E predominates in the lung tissue from both IPF patients and BLM‐induced lung fibrosis mice, we aimed to elucidate which Sema3E form is the main pro‐fibrotic active form of Sema3E underlying IPF. As Sema3E functions as a secreted protein, we employed recombinant P87‐Sema3E and P61‐Sema3E proteins for subsequent experiments. Initially, we verified the molecular weights of these recombinant proteins using western blot analysis (**Figure** **4**A). Subsequently, we treated fibroblasts derived from patients with IPF with various concentrations of P61‐Sema3E and P87‐Sema3E recombinant proteins. Notably, P61‐Sema3E, but not P87‐Sema3E, significantly upregulated the protein expression of Fibronectin, Col1a1, and α‐SMA in IPF fibroblasts in a concentration‐dependent manner (Figure 4B, Figure S2A). Furthermore, EdU assays showed that P61‐Sema3E promoted the proliferation of primary human lung fibroblasts in a concentration‐dependent manner (Figure 4C). Similarly, in transwell assays, P61‐Sema3E demonstrated a concentration‐dependent promotion of primary human lung fibroblast migration (Figure 4D).

To ascertain whether the effects of P61‐Sema3E are mediated through Plexin D1, we stimulated cells with P61‐Sema3E following Plexin D1 knockdown in primary human lung fibroblasts. The results revealed that the pro‐fibrotic effect of P61‐Sema3E was mitigated after Plexin D1 knockdown (Figure 4E). Moreover, the absence of Plexin D1 in primary human lung fibroblasts inhibited the migration and proliferation induced by P61‐Sema3E (Figure 4F,G).

In summary, our findings indicate that P61‐Sema3E serves as a major active component in promoting fibroblast activation, proliferation, and migration. Additionally, P61‐Sema3E exerts its effects through the Plexin D1 receptor.

### 2.5 P61‐Sema3E Produces a Pro‐Fibrotic Effect through the Activation of ErbB2

Given that TGF‐β1 is a key driver of fibrosis, primarily acting through Smad2/3 phosphorylation, we hypothesized that the Sema3E–Plexin D1 axis might influence TGF‐β1 mediated Smad signaling. To investigate this, we conducted experiments using primary lung fibroblasts stimulated with varying concentrations of P87‐Sema3E and P61‐Sema3E. Western blot analysis was performed to assess levels of phosphorylated Smad2 (p‐Smad2) and phosphorylated Smad3 (p‐Smad3). Our results demonstrated that neither P87‐Sema3E nor P61‐Sema3E significantly increased p‐Smad2 or p‐Smad3 levels under these conditions (Figure S3, Supporting Information).

**Figure 5:**
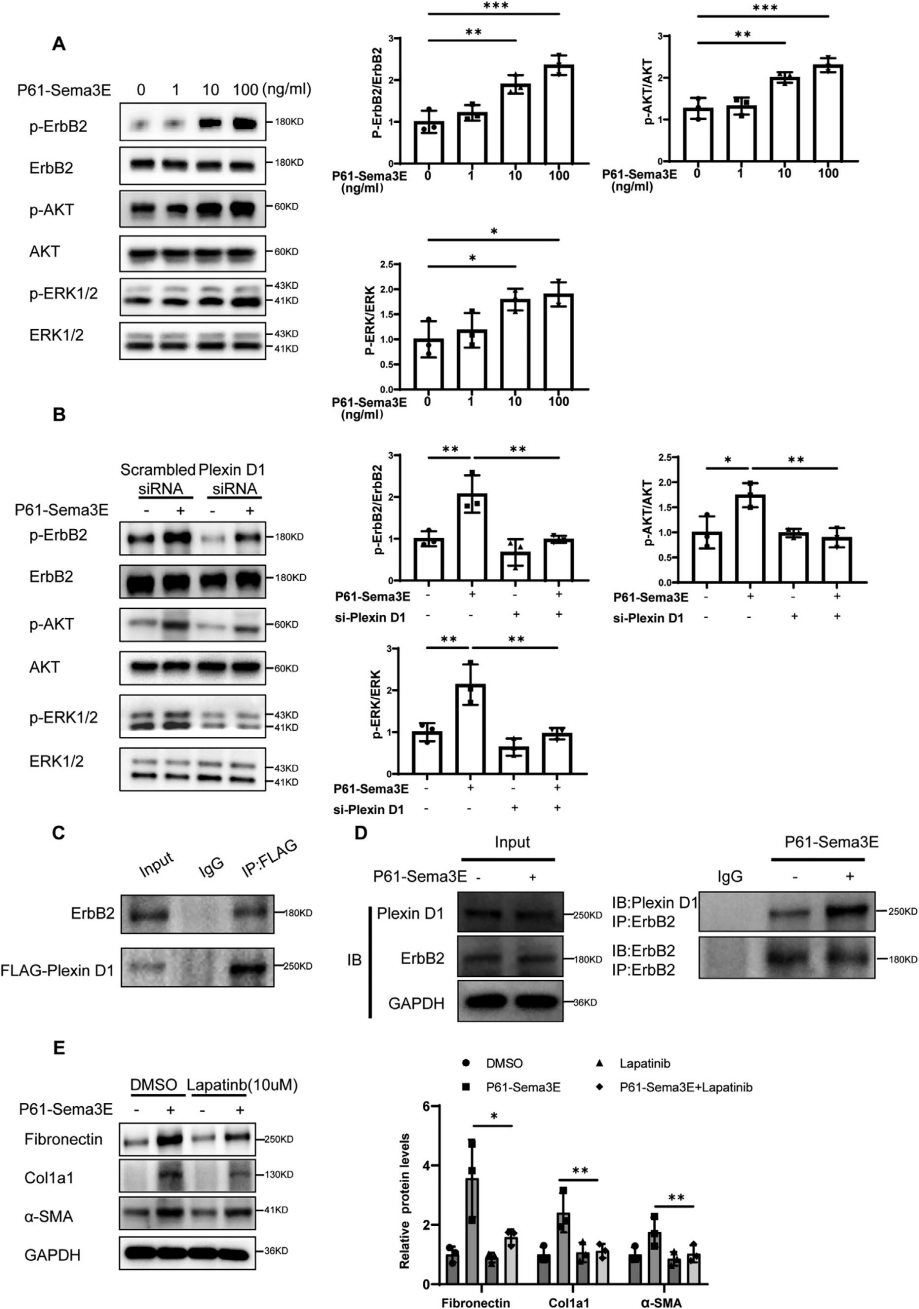
P61‐Sema3E produces a pro‐fibrotic effect through the activation of ErbB2. A) Western blot analysis of the levels of ErbB2, P‐ErbB2, AKT, P‐AKT, ERK1/2, and P‐ERK1/2 in primary human lung fibroblasts (PHLFs) after stimulation with different concentrations of P61‐Sema3E for 2 h. B) Western blot analysis of ErbB2, P‐ErbB2, AKT, P‐AKT, ERK1/2, and P‐ERK1/2 expression in PHLFs treated with PlexinD1 siRNA or Scrambled siRNA following P61‐Sema3E induction. C) Co‐immunoprecipitation analysis of Plexin D1 and ErbB2, FLAG‐tagged Plexin D1 plasmid were transfected into PHLFs. D) Co‐immunoprecipitation analysis of Plexin D1 and ErbB2, PHLFs were treated with P61‐Sema3E for 2 h. E) Western blot analysis of Fibronectin, Col1a1, and α‐SMA expression in PHLFs treated with the ErbB2 inhibitor Lapatinib or PBS following P61‐Sema3E stimulation for 48 h. Data are represented as the mean ± SEM of three independent experiments. Statistical analyses were performed using one‐way ANOVA test. *p < 0.05; **p < 0.01; ***p < 0.001.

We considered the possibility that the Sema3E‐Plexin D1 axis might not mediate fibrosis through the common fibrotic pathways. Interestingly, in tumors, the ErbB2 signaling pathway has been reported to associate with P61‐Sema3E, suggesting a potential alternative mechanism,^[^ ^13^ ^]^ However, it remains unknown whether ErbB2 mediates P61‐Sema3E‐related biological processes in fibroblasts. To explore this, primary human lung fibroblasts were treated with varying concentrations of P61‐Sema3E recombinant protein for 2 h. Remarkably, we observed that P61‐Sema3E stimulation led to the promotion of ErbB2 phosphorylation. Western blot analysis revealed a significant concentration‐dependent upregulation of the p‐ErbB2/ErbB2 ratio following P61‐Sema3E stimulation (**Figure** **5**A). Because MAPK and PI3K‐AKT are major players in studies related to ErbB2‐mediated signaling pathways, we also assayed the phosphorylation of Erk1/2 and AKT, Consistently, western blot results showed p‐AKT/AKT ratio and p‐ERK/ERK ratios were all enhanced in a concentration‐dependent manner upon stimulation with different concentrations of P61‐Sema3E (Figure 5A). Next, to demonstrate that the effect of P61‐Sema3E on ErbB2 phosphorylation was mediated by Plexin D1. We used si‐Plexin D1 to knock down the expression of Plexin D1 in primary human lung fibroblasts and stimulated the primary human lung fibroblasts with P61‐Sema3E. Western blot results showed that Plexin D1 knockdown inhibited ErbB2 phosphorylation induced by P61‐Sema3E in primary human lung fibroblasts (Figure 5B). Consistently, the phosphorylation of the AKT and ERK pathways downstream of ErbB2 induced by P61‐Sema3E was also inhibited by the application of si‐Plexin D1 (Figure 5B). Next, to investigate the interaction between Plexin D1 and ErbB2, we utilized a Flag‐tagged plasmid to overexpress Plexin D1 in fibroblasts and conducted co‐immunoprecipitation (co‐IP) experiments. Notably, we successfully co‐purified Plexin D1 and ErbB2 from the lysates of cells overexpressing Plexin D1. This finding suggests that Plexin D1 and ErbB2 can form receptor complexes on the surface of fibroblasts (Figure 5C). In addition, we conducted endogenous co‐IP to isolate ErbB2 from fibroblasts. Our results demonstrated that the interaction between the endogenous receptors Plexin D1 and ErbB2 was significantly enhanced upon stimulation with P61‐Sema3E (Figure 5D). It is suggested that they can form receptor complexes on the cell surface, P61‐Sema3E can promote the phosphorylation of ErbB2 via the Plexin D1 receptor, consequently activating the ErbB2 pathway. To confirm that the pro‐fibrotic function of P61‐Sema3E operates through the phosphorylation of ErbB2, we pre‐treated primary human lung fibroblasts with the ErbB2 inhibitor lapatinib for 2 h, followed by stimulation with P61‐Sema3E for 48 h. Our results demonstrate that the P61‐Sema3E‐induced activation of primary human lung fibroblasts could indeed be suppressed by ErbB2 inhibitors. Western blot analysis showed a notable reduction in the expression levels of Fibronectin, Col1a1, and α‐SMA induced by P61‐Sema3E stimulation upon treatment with ErbB2 inhibitor lapatinib (Figure 5E).

Moreover, our findings indicate that the P61‐Sema3E‐induced proliferation and migration of primary human lung fibroblasts were also inhibited by ErbB2 inhibitor lapatinib, as evidenced by EDU experiments and transwell assays (Figure S4A,B, Supporting Information). These results provide compelling evidence that the pro‐fibrotic effects of P61‐Sema3E are indeed mediated through the phosphorylation of ErbB2, thereby elucidating a crucial mechanistic pathway underlying the pathogenesis of pulmonary fibrosis.

### 2.6 Silencing Sema3E Alleviated Experimental Pulmonary Fibrosis in Mice

**Figure 6:**
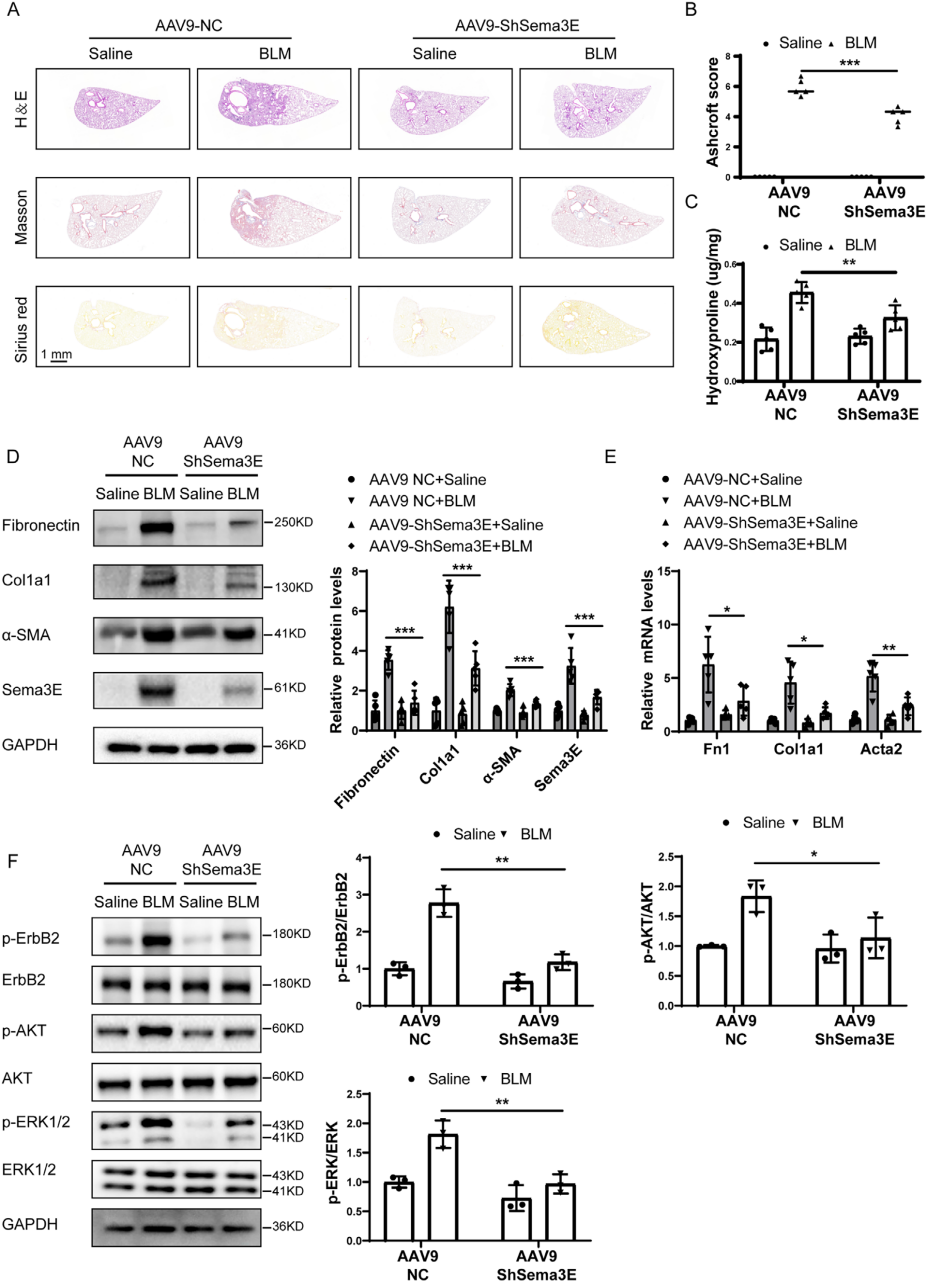
Downregulation of Sema3E attenuates BLM‐induced pulmonary fibrosis. A,B) Histological analysis of lung fibrosis severity in mice following BLM induction. (A) Representative images of lung sections stained with H&E, Masson's trichrome, and Sirius red to assess fibrosis severity. (B) Bar graph showing the quantitative mean score of fibrosis severity. Samples include AAV9‐NC saline mice (*n* = 5), AAV9‐ShSema3E saline mice (*n* = 5), AAV9‐NC BLM mice (*n* = 5), and AAV9‐ShSema3E BLM mice (*n* = 5). C) Quantification of hydroxyproline contents in AAV9‐NC mice and AAV9‐ShSema3E mice after BLM challenge. D,E) Western blot and RT‐qPCR analysis of Fibronectin, Col1a1, and α‐SMA protein and mRNA expression in lung homogenates from the mentioned mouse groups. F) Western blot analysis of ErbB2, P‐ErbB2, AKT, P‐AKT, ERK1/2, and P‐ERK1/2 expression in lung homogenates from the mentioned mouse groups. Samples include AAV9‐NC saline mice (*n* = 3), AAV9‐ShSema3E saline mice (*n* = 3), AAV9‐NC BLM mice (*n* = 3), and AAV9‐ShSema3E BLM mice (*n* = 3). Data are represented as the mean ± SEM. Statistical analyses were performed using one‐way ANOVA tests. *p < 0.05; **p < 0.01; ***p < 0.001.

Continuing our investigation into the comprehensive role of Sema3E in BLM‐induced pulmonary fibrosis, we sought to determine whether suppression of Sema3E could mitigate pulmonary fibrosis in mice. To achieve this, we utilized adenovirus‐associated virus 9 (AAV9) carrying a shSema3E fragment, designated as AAV9‐shSema3E. Fourteen days prior to BLM administration, AAV9‐shSema3E was intrabronchially injected into mice, subsequently, lung fibrosis was assessed 21 days after BLM induction. Western blot and immunofluorescence analyses showed that Sema3E expression was significantly reduced in the lung tissues of AAV9‐shSema3E mice compared to controls, including in fibroblasts (Figure S5A,B, Supporting Information). Hematoxylin and eosin (H&E), Masson's trichrome staining, and Sirius red staining of lung sections from the BLM group showed a significant increase in fibrotic lesions in the lung parenchyma compared to the saline group, and AAV9‐shSema3E group effectively ameliorated BLM‐induced lung parenchymal fibrotic lesions compared with AAV9‐NC group, as evidenced by lower Ashcroft scores (**Figure** **6**A,B). Consistently, significantly lower levels of hydroxyproline, a major component of fibrillar collagen of all types, were also detected in the AAV9‐shSema3E group mice (Figure 6C). Furthermore, we assessed the expression of fibrotic markers expression through western blot analysis and RT‐qPCR. Results showed that mice treated with AAV9‐shSema3E exhibited lower protein and mRNA levels of Fibronectin, Col1a1, and α‐SMA expression following BLM‐induced pulmonary fibrosis compared to AAV9‐NC mice (Figure 6D,E).

Moreover, we evaluated the levels of p‐ErbB2 and ErbB2 in the lung homogenates derived from AAV9‐NC and AAV9‐shSema3E mice following BLM induction. Consistently, the ratios of p‐ErbB2/ErbB2 were found to increase in the lung homogenates from BLM‐induced mice compared to control mice (Figure 6F). However, the loss of Sema3E largely reversed these changes in the levels of ErbB2 and p‐ErbB2 induced by BLM. Similarly, downstream signaling pathways such as AKT and ERK1/2 exhibited trends consistent with the ErbB2 pathway (Figure 6F).

Taken together, our findings indicate that the absence of Sema3E confers protection against BLM‐induced lung fibrosis in mice, highlighting the potential therapeutic significance of targeting Sema3E in the treatment of pulmonary fibrosis.

### 2.7 Sema3E Deficiency in Fibroblasts Protects Mice from BLM‐Induced Lung Injury and Fibrosis

**Figure 7:**
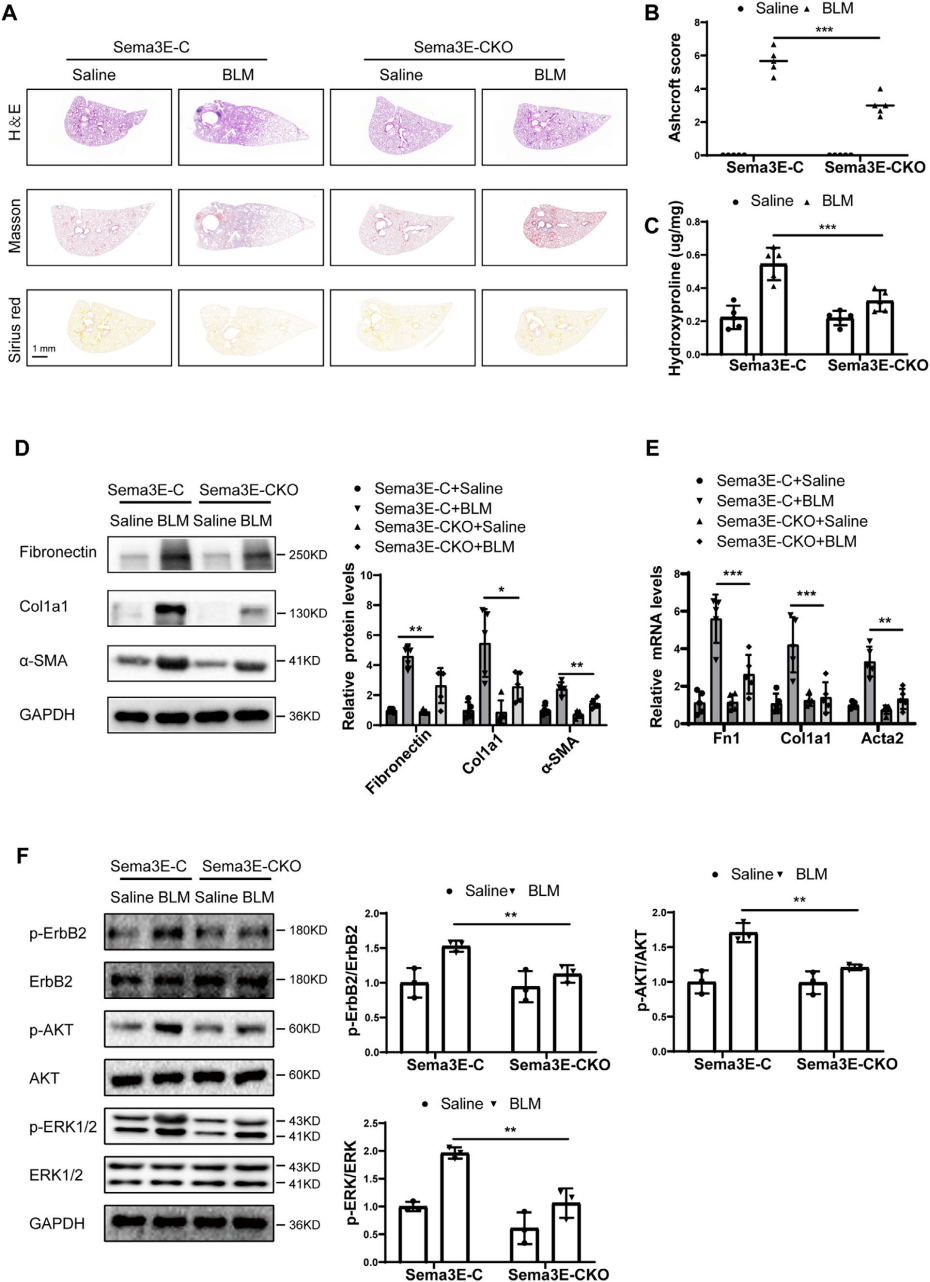
Sema3E deficiency in fibroblasts protects mice from BLM‐induced lung injury and fibrosis. A,B) Representative lung sections from Sema3E‐C and Sema3E‐CKO mice treated with saline or BLM, stained with H&E, Masson's Trichrome, and Sirius Red (Panel A, left). Panel B (right) shows quantitative fibrosis scores. Each group consisted of five mice (*n* = 5). C) Quantification of hydroxyproline contents in Sema3E‐CKO and Sema3E‐C mice after BLM challenge. D,E) Western blot and RT‐qPCR analyses of Fibronectin, Col1a1, and α‐SMA protein and mRNA levels in lung homogenates from each group (*n* = 5). F) Western blot analysis of ErbB2, P‐ErbB2, AKT, P‐AKT, ERK1/2, and P‐ERK1/2 in lung homogenates from the same groups of three mice(*n* = 3). Data are expressed as the mean ± SEM. Statistical significance was determined by one‐way ANOVA (*p < 0.05; **p < 0.01; ***p < 0.001).

Previous in vitro studies have shown that Sema3E knockdown suppresses fibroblast activation, proliferation, and migration, while in vivo knockdown of Sema3E in whole lung tissue using AAV9‐shSema3E significantly alleviates lung fibrosis. These findings suggest that the protective effect of Sema3E knockdown in fibrosis primarily results from its reduction in fibroblasts. To further investigate the role of Sema3E in fibroblasts in vivo, we created fibroblast‐specific Sema3E knockout mice, denoted as Col1α2‐Cre^+^ Sema3E flox/flox (Sema3E‐CKO), using the method illustrated in Figure S6A (Supporting Information). Co‐immunostaining of lung sections demonstrated the absence of Sema3E in fibroblasts from the Sema3E‐CKO group compared with the Sema3E‐C (control) group (Figure S6C, Supporting Information). These findings were corroborated by genotyping assays conducted on DNA extracted from mice tissues (Figure S6D, Supporting Information). Additionally, Western blot analysis confirmed a marked reduction in Sema3E protein levels (Figure S6B, Supporting Information). Both Sema3E‐C and Sema3E‐CKO mice were administered BLM intratracheally at a dose of 0.5 mg kg^−1^ to induce lung injury and fibrosis. Mice were monitored for 21 days, after which lung tissues were harvested for histological and molecular analyses. And histological analyses (H&E, Masson's trichrome, and Sirius Red staining) revealed significantly reduced fibrotic changes in the Sema3E‐CKO group, accompanied by a markedly lower Ashcroft score when compared to control mice (**Figure** **7**A,B). Additionally, significantly lower hydroxyproline levels were observed in the Sema3E‐CKO group (Figure 7C). Moreover, Western blot and RT‐qPCR analyses demonstrated a significant reduction in both protein and mRNA levels of fibrosis markers, including Fibronectin, Col1a1, and α‐SMA, in the lung tissues of Sema3E‐CKO mice compared to the control group (Figure 7D,E). These results indicate that Sema3E plays a crucial role in facilitating fibroblast‐driven fibrosis in the lung, and that its targeted deletion in fibroblasts confers significant protection against BLM‐induced pulmonary fibrosis.

To explore the underlying mechanisms, we assessed the ErbB2 signaling pathway and its downstream activation of ERK and AKT. Consistent with earlier findings, fibroblast‐specific knockout of Sema3E significantly inhibited the hyperactivation of ErbB2, ERK, and AKT induced by BLM exposure (Figure 7F). These results provide strong evidence that Sema3E plays a central role in mediating fibroblast‐driven lung fibrosis via ErbB2‐dependent pathways.

### 2.8 Therapeutic Effects of Using Furin Inhibitors in IPF

**Figure 8:**
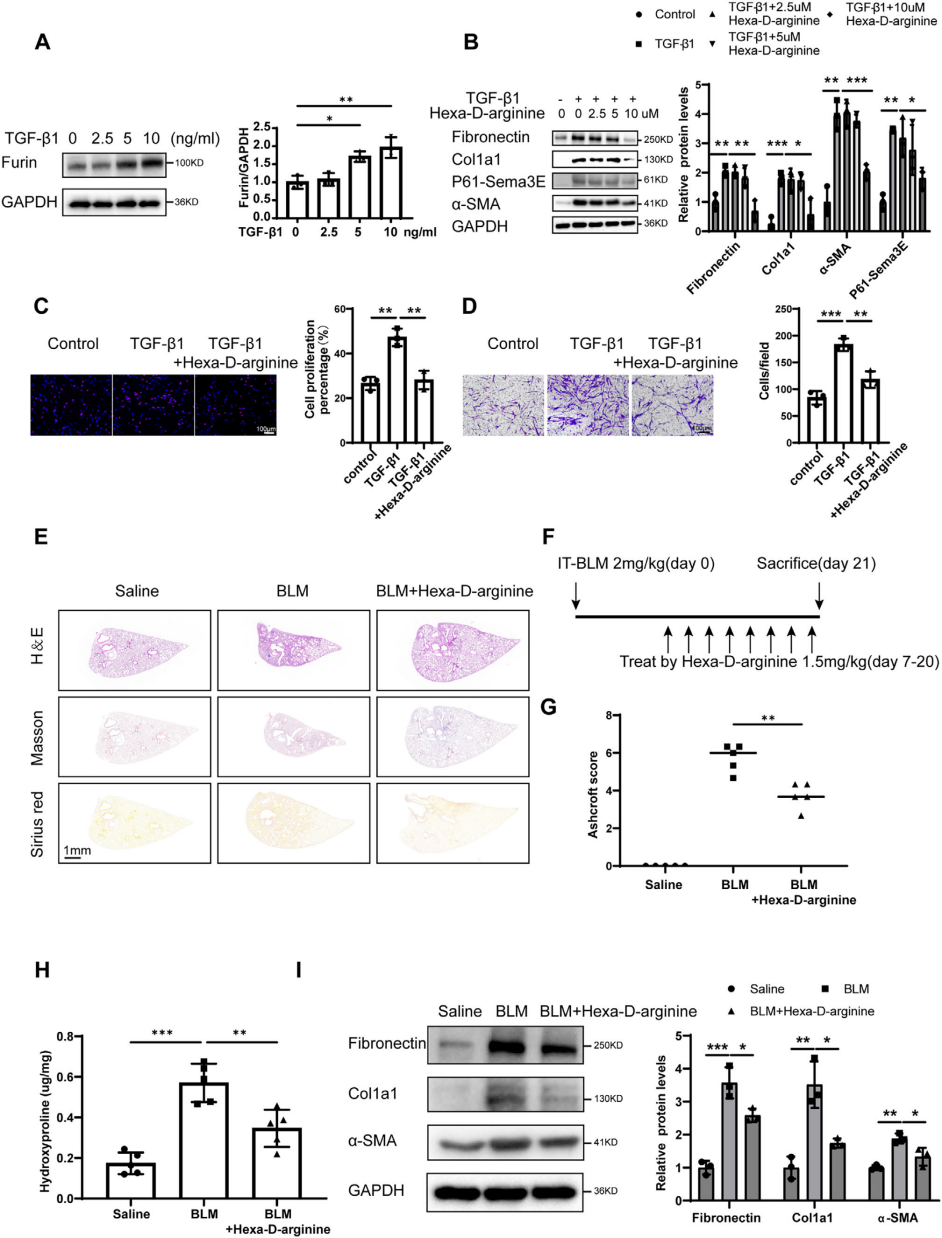
Furin inhibitor suppresses differentiation, proliferation, and migration of fibroblasts in IPF. A) Western blot analysis of Furin levels in primary human lung fibroblasts (PHLFs) exposed to varying concentrations of TGF‐β1 for 48 h. B) Western blot analysis of P61‐Sema3E, Fibronectin, Col1a1, and α‐SMA expression in PHLFs treated with different concentrations of the Furin inhibitor Hexa‐D‐arginine following TGF‐β1 induction for 48 h. C) Representative images of EdU staining in PHLFs treated with Hexa‐D‐arginine after TGF‐β1 induction for 48 h. D) Representative images from the Transwell assay in PHLFs treated with Hexa‐D‐arginine following TGF‐β1 induction for 48 h. Images were captured at ×200 magnification. E,G) Histological assessment of lung fibrosis severity in mice following BLM induction. (E) Left panel: Representative lung sections stained with H&E, Masson's trichrome, and Sirius Red to evaluate fibrosis severity. (G) Right panel: Bar graph quantifying fibrosis severity scores (Saline: *n* = 5, BLM: *n* = 5, BLM + Hexa‐D‐arginine: *n* = 5). F) Schematic representation of the experimental design. Mice received intratracheal instillation of 2 mg kg^−1^ BLM and were administered intraperitoneal injections of Hexa‐D‐arginine starting on day 7 until the end of the experiment. H) Quantification of hydroxyproline content in lung tissues. I) Western blot analysis of Fibronectin, Col1a1, and α‐SMA protein expression in lung homogenates from the indicated mouse groups (Saline: *n* = 5, BLM: *n* = 5, BLM + Hexa‐D‐arginine: *n* = 5). Data are presented as the mean ± SEM from three independent experiments. Statistical significance was determined using one‐way ANOVA (*p < 0.05; **p < 0.01; ***p < 0.001).

Sema3E is first produced as a full‐length precursor with a molecular weight of ≈87 kDa (p87‐Sema3E). This precursor undergoes maturation through proteolytic cleavage by Furin, resulting in a smaller fragment of ≈61 kDa, known as p61‐Sema3E. Given that the production of P61‐Sema3E is mediated by the function of Furin. Next, we conducted experiments to determine whether the increased expression of P61‐Sema3E in IPF was attributable to changes in Furin expression. We conducted western blot analysis to investigate the alterations in Furin expression in fibroblasts following TGF‐β1 stimulation, and demonstrated that TGF‐β1 induced Furin expression in a dose‐dependent manner (**Figure** **8**A). Further analysis was conducted to assess the expression of Furin in lung tissues obtained from both IPF patients and mice with BLM‐induced pulmonary fibrosis and confirmed that Furin expression was up‐regulated in IPF (Figure S7A,B, Supporting Information). Subsequently, primary human lung fibroblasts were treated with different concentration of Furin inhibitor Hexa‐D‐arginine followed by TGF‐β1 stimulation. Through western blot analysis, we demonstrated that treatment with Furin inhibitor led to a significant downregulation of P61‐Sema3E expression in primary human lung fibroblasts (Figure 8B). These findings collectively suggest that TGF‐β1 stimulation leads to an increase in Furin expression, which subsequently induces elevated levels of P61‐Sema3E expression in fibroblasts.

Our findings corroborate that P61‐Sema3E plays a pivotal role in promoting fibrosis, while no comparable function was observed for P87‐Sema3E. Consequently, targeting Furin with inhibitors may offer a viable approach to impede the binding of Plexin D1 and P61‐Sema3E by preventing the cleavage of P87‐Sema3E into its pro‐fibrotic form, P61‐Sema3E. Next, to assess the impact of Furin inhibitors on fibroblast activation, we treated primary human lung fibroblasts with varying concentrations of Furin inhibitor Hexa‐D‐arginine subsequent to TGF‐β1 induction. Western blot analysis revealed a concentration‐dependent decrease in the expression levels of Fibronectin, Col1a1, and α‐SMA following Furin inhibitor treatment (Figure 8B). Furthermore, through EDU experiments, we demonstrated that Furin inhibition attenuated TGF‐β1‐induced fibroblast overproliferation (Figure 8C). Consistently, in transwell assays, we observed that the Furin inhibitor Hexa‐D‐arginine suppressed TGF‐β1‐induced fibroblast overmigration (Figure 8D).

Based on the above observations, we aimed to investigate whether Furin also exerts a therapeutic effect in vivo. We induced pulmonary fibrosis in mice and administered Hexa‐D‐arginine (1.5 mg kg^−1^) intravenously every other day, starting on the 14th day of modeling (Figure 8F). Histological analyses, including H&E, Masson Trichrome, and Sirius Red staining, revealed a significant reduction in pulmonary fibrosis in Hexa‐D‐arginine‐treated mice (Figure 8E,G). Additionally, similar results were obtained in the levels of hydroxyproline (Figure 8H). Likewise, mice administered Hexa‐D‐arginine exhibited a notable decrease in fibrosis markers at the protein level (Figure 8I).

These findings collectively underscore the potential therapeutic efficacy of Furin inhibitors in mitigating fibroblast activation, proliferation, and migration, highlighting their promising role in combating pulmonary fibrosis.

## 3 Discussion

The complex etiology of IPF and the lack of effective therapeutic approaches pose significant challenges to clinical management. Thus, a thorough understanding of the factors contributing to the pathogenesis of IPF is crucial. Although Sema3E exhibits diverse pathophysiological functions in various diseases, our study provides direct experimental evidence implicating Sema3E in the pathogenesis of pulmonary fibrosis. Our research demonstrated that plasma levels of Sema3E were significantly elevated in patients with IPF compared to controls. Similar findings were observed in BLM‐induced pulmonary fibrosis mice. Notably, Sema3E predominantly appeared as P61‐Sema3E in the plasma and lung tissues of IPF patients, as well as in lung homogenates from fibrosis‐induced mice. Mechanically, TGF‐β1 induced Furin expression in fibroblasts, which in turn promoted P61‐Sema3E expression. P61‐Sema3E facilitated the phosphorylation of ErbB2 through Plexin D1 binding, subsequently activating the AKT and ERK1/2 pathways and thereby regulating fibroblast differentiation, proliferation, and migration.

Our study demonstrated elevated expression of both Sema3E in lung tissue and plasma samples from IPF patients, studies in systemic sclerosis have similarly shown increased Sema3E expression in the serum of patients with systemic sclerosis patients, corroborating our findings. However, it's important to note that while our study implicates Sema3E in the pathogenesis of pulmonary fibrosis, previous studies have reported contrasting findings in lung studies of Sema3E. Specifically, investigations have revealed significant downregulation of Sema3E expression in the bronchoalveolar lavage fluid and lung tissues of asthma patients compared to normal subjects. Additionally, in animal experiments modeling asthma, administration of full‐length P87‐Sema3E was found to inhibit lung airway remodeling.^[^ ^22^, ^23^ ^]^ The discrepancy observed between our findings regarding Sema3E in pulmonary fibrosis and previous studies in asthma prompted us to delve deeper into understanding the underlying mechanisms. Specifically, we investigated the two forms of Sema3E: full‐length Semaphorin3E (p87‐Sema3E) and the protein hydrolyzed fragment (p)61 kDa Sema3E (p61‐Sema3E). Our investigation has unveiled a clear divergence in the pattern of Sema3E form expression between pulmonary fibrosis and asthma. In the plasma and lung tissue of IPF patients, we predominantly detected P61‐Sema3E, alongside lower expression levels of P87‐Sema3E, with no significant differences observed between groups. Conversely, in asthma, the diminished Sema3E levels were primarily attributed to a reduction in P87‐Sema3E levels.^[^ ^22^, ^24^ ^]^ This observed contrast in Sema3E form expression between pulmonary fibrosis and asthma could indeed be attributed to disparities in Furin expression. Intriguingly, our investigation unveiled significantly elevated Furin levels in IPF, potentially contributing to the prevalent presence of P61‐Sema3E in this condition. In contrast, no significant difference in Furin expression was observed in bronchial brush samples from asthmatic patients compared to healthy subjects.^[^ ^25^ ^]^ Furthermore, it's notable that Furin expression is notably upregulated in tumors.^[^ ^26^, ^27^ ^]^ Our findings align with previous research on Sema3E‐PlexinD1 function in tumors, indicating that P61‐Sema3E is the predominant form found in invasive and metastatic cancer cells.^[^ ^13^ ^]^

The mechanism underlying the increased expression of Furin in IPF remains unclear. Studies in human granulosa‐lutein (hGL) cells have shown that TGF‐β1 stimulation induces upregulation of Furin expression through TβRII.^[^ ^28^ ^]^ We observed a similar phenomenon in fibroblasts, where Furin expression exhibited a concentration‐dependent response to TGF‐β1 stimulation. However, the precise underlying mechanisms require further investigation.

In IPF, fibroblasts exhibit unregulated proliferation, migration, and differentiation into myofibroblasts which overproduce ECM components.^[^ ^29^, ^30^ ^]^ We further established that P61‐Sema3E is the predominantly active form that promotes lung fibrosis, as evidenced by stimulating fibroblasts with recombinant proteins of different Sema3E forms. Additionally, we confirmed that P61‐Sema3E, like P87‐Sema3E, acts through the Plexin D1 receptor. Collectively, our results indicate that fibroblasts secrete P61‐Sema3E in response to TGF‐β1 stimulation. P61‐Sema3E then interacts with the Plexin D1 receptor on the fibroblast cell membrane through autocrine signaling, promoting fibroblast proliferation, migration, and differentiation.

The previous report showed that Furin inhibitors reduce EMT by inhibiting the cleavage of full‐length Sema3E to P61‐Sema3E, which in turn reduces the invasive and migratory capacity of Colorectal cancer.^[^ ^15^ ^]^ Since P61‐Sema3E is the main active form of pro‐fibrotic, we tested whether Furin inhibitors has an anti‐fibrotic effect. Initially, we verified Furin's pivotal role in catalyzing the cleavage of P87‐Sema3E into P61‐Sema3E within fibroblasts. Furthermore, we demonstrated that Furin inhibitors effectively attenuated TGF‐β1‐induced fibroblast proliferation, migration, and differentiation by impeding the generation of P61‐Sema3E. Notably, the treatment of mice with Furin inhibitor Hexa‐D‐arginine strikingly reversed the pulmonary fibrosis.

Next, we investigated how the P61‐Sema3E–Plexin D1 axis promotes fibroblast proliferation, migration, and differentiation. First, we examined whether Sema3E influences the typical TGF‐β pathway. Under our experimental conditions, neither P87‐Sema3E nor P61‐Sema3E significantly increased p‐Smad2 or p‐Smad3 levels (Figure S3, Supporting Information). These findings suggest that Sema3E's pro‐tissue necrotic effects occur via mechanisms other than the classical TGF‐β–Smad axis. We therefore explored alternate pathways and noted that previous studies reported an interaction between Plexin D1 and ErbB2.^[^ ^13^ ^]^ ErbB2 receptor signaling has been reported to regulate cell proliferation, migration, differentiation, apoptosis, and cellular movement through the AKT and ERK pathways.^[^ ^31^ ^]^ And it has been suggested that ErbB2 is involved in the regulation of fibroblast invasion and lung fibrosis.^[^ ^32^ ^]^ Furthermore, it has been reported that the AKT and ERK pathways promote fibroblast proliferation, differentiation, and migration.^[^ ^33^, ^34^, ^35^ ^]^ Therefore, we stimulated fibroblasts with various concentrations of P61‐Sema3E to assess ErbB2 phosphorylation and the activation of downstream AKT and ERK pathways. Our observations indicated that P61‐Sema3E activated ErbB2 phosphorylation, which subsequently led to the activation of the downstream AKT and ERK pathways in these cells. Additionally, the knockdown of Plexin D1 expression in fibroblasts inhibited the P61‐Sema3E‐induced activation of the ErbB2, AKT, and ERK pathways. This finding suggests that P61‐Sema3E may facilitate the activation of the ErbB2 pathway via the receptor Plexin D1. To investigate the mechanism by which P61‐Sema3E promotes ErbB2 activation via Plexin D1, we assessed the interaction between Plexin D1 and ErbB2 using a co‐IP assay. Interestingly, we found a binding interaction between Plexin D1 and ErbB2 in fibroblasts to form a receptor complex on the cell surface. Furthermore, we found that P61‐Sema3E enhanced the binding of Plexin D1 to ErbB2. Thus, P61‐Sema3E may promote phosphorylation of ErbB2 by facilitating the binding of Plexin D1 to ErbB2. It was observed that Plexin D1 facilitates ErbB2 phosphorylation in tumors, aligning with our findings and reinforcing the interconnectedness of these pathways.^[^ ^13^ ^]^ Additionally, to further confirm that P61‐Sema3E influences ErbB2 phosphorylation via Plexin D1, affecting fibroblast proliferation, migration, and activation, we inhibited ErbB2 activity using a specific inhibitor Lapatinb. Our results indicated that ErbB2 inhibition reduced the proliferation, migration, and activation of fibroblasts induced by P61‐Sema3E. Overall, our data demonstrate that the P61‐Sema3E‐Plexin D1 promotes fibroblast proliferation, migration, and differentiation by enhancing ErbB2 phosphorylation and stimulating the activation of the AKT and ERK pathways.

Our study has some limitations. Knocking down Sema3E reduced both P87‐Sema3E and P61‐Sema3E, limiting the assessment of their distinct roles. While P87‐Sema3E lacks direct fibrosis‐promoting activity, it may indirectly affect fibroblast function. Isoform‐specific approaches are needed in future studies to clarify their contributions. And we focused on the autocrine role of Sema3E in fibroblasts, but Sema3E, may be secreted to other cells of the IPF and affect other cells function as paracrine, which needs to be further investigated. Furthermore, Furin can also participate in post‐translational shearing of other proteins in addition to affecting full‐length Sema3E shearing, treating IPF with Furin inhibitors might lack precision.

In conclusion, our study revealed upregulation of Sema3E expression in both plasma and lung tissues of IPF patients, with a notable correlation observed with lung function indices. Furthermore, we identified that Sema3E predominantly exists in the form of P61‐Sema3E in the lung tissue and blood of IPF patients. Subsequent investigations indicated that the increased expression of P61‐Sema3E in IPF is attributed to the upregulation of Furin expression in the lung tissues of IPF patients. Importantly, we demonstrated that elevated P61‐Sema3E levels promote the proliferation, migration, and differentiation of fibroblasts, implicating its involvement in the pathological processes underlying IPF. These findings offer valuable insights into the mechanisms underlying pulmonary fibrosis, and indicate that targeting Sema3E could be a promising approach for IPF treatment. Moreover, inhibiting the production of P61‐Sema3E using Furin inhibitors may also hold promise for IPF therapy. In addition, Sema3E has the potential to be used as a plasma biomarker for the clinical diagnosis of IPF.

## 4 Experimental Section

### Clinical Subjects

Plasma samples were collected from 63 IPF patients and 24 control subjects, whereas lung tissue samples were obtained from 12 IPF patients and 10 control subjects. All samples were provided by Tongji Hospital in Wuhan, between 2021 and 2024. All lung specimens were obtained from patients who underwent lung transplantation or surgical resection of lung masses in thoracic surgery. The diagnosis of IPF was based on the consensus criteria established by the European Respiratory Society (ERS) and the American Thoracic Society (ATS). Detailed patient information is provided in Tables S1 and S2 (Supporting Information).

### Primary Human Lung Fibroblast Isolation Culture, And Treatment

In our experiments, fibroblasts were obtained from three distinct sources, each isolated from different donors. Lung tissue samples were obtained from human surgical specimens, and excess blood was removed by PBS perfusion. Tissue fragments were prepared in PBS and cultured in 2 mL of DMEM complete medium (10% fetal bovine serum, 1% penicillin/streptomycin) in a 10‐cm dish, ensuring adherence without floating or clumping. After 24 h, 8 mL of fresh medium was added, and non‐adherent cells were removed after three days through washing or medium replacement. Spindle‐shaped cells migrated from the tissue fragments, and outgrowth covered the dish within six days, enabling passaging.

Over successive passages, fibroblasts dominated the culture, while epithelial cells and macrophages were eliminated. Passage 4 fibroblasts were used for experiments. Lung fibroblasts were identified by S100A4 immunofluorescence staining.

In knockdown experiment, siRNA specific for Sema3E(5′‐GCTGGACTCTACAGTGACT‐3′) and Plexin D1(5′‐ GGAUAUGUCUGGAUAGAAATT‐3′), were procured from RiboBio (Guangzhou, China) and DesignGene (Shanghai, China). Primary human lung fibroblasts were transiently transfected using Lipofectamine 3000 (Invitrogen, Shanghai, China).

The ErbB2 inhibitor Lapatinib, was obtained from MedChemExpress (CM00323). For experiments, Lapatinib was diluted in culture medium to a final concentration of 10 µM. Cells were pre‐treated with Lapatinib for 2 h before stimulation with P61‐Sema3E.

The Furin inhibitor Hexa‐D‐arginine (HY‐P1028, MedChemExpress, China) was obtained from MedChemExpress. Cells were pre‐treated with Hexa‐D‐arginine for 2 h prior to TGF‐β1 stimulation.

### Plasmids and Transfection

Flag‐Plexin D1 and control plasmids were obtained from Designgene Biotechnology (Shanghai, China). Briefly, fibroblasts were seeded in 10‐cm dishes after passaging and grown to ≈70–80% confluence. Cells were then transiently transfected with either the Flag‐Plexin D1 plasmid or the control plasmid using Lipofectamine P3000 reagent (Thermo Fisher Scientific, USA). To prepare the transfection mixture, 10 µg of the purified DNA was combined with transfection reagent before being added to the cells. After a 48‐h incubation, cells were harvested and lysed in NP‐40 buffer for subsequent co‐IP experiments.

### Antibodies and Reagents

Antibodies for Sema3E (AF3239, R&D Systems, USA), Plexin D1(AF4160, R&D Systems, USA), Fibronectin(15613‐1‐AP, Proteintech, China), Collagen Type I(66761‐1, Proteintech, China), α‐SMA(119 245, CST, USA), Phospho‐HER2/ErbB2 (2243, CST, USA), Phospho‐AKT(4060, CST, USA), Phospho‐p44/42 MAPK(4370, CST, USA), ErbB2(18299‐1‐AP, Proteintech, China), AKT (4691, CST, USA), p44/42 MAPK(4695, CST, USA), GADPH(AC001, Abclonal, China), Phospho‐SMAD2(# 8828, CST, USA), Phospho‐SMAD3(#9520, CST, USA), SMAD2/3(#3102, CST, USA). Recombinant P61‐Sema3E were bought from Cloud‐Clone company(RPL920Hu01, Cloud‐Clone, China), Recombinant P87‐Sema3E were bought from R&D Systems(3239‐S3B, R&D Systems, USA), TGFβ1 were bought from MedChemExpress company(HY‐P7118, MCE, USA), Hexa‐D‐arginine were bought from MedChemExpress company(HY‐P1028, MCE, USA).

### Real‐Time Quantitative PCR

Total RNA was extracted from fibroblasts or tissues using TRIzol reagent (Takara Bio, Japan) following the manufacturer's protocols. Reverse transcription was performed using the HiScript III 1st Strand cDNA Synthesis Kit (Vazyme, China). Subsequently, RT‐qPCR was performed employing the ChamQ Universal SYBR qPCR Master Mix (Vazyme, China). Refer to Table S3 (Supporting Information) for more detailed primer information.

### Immunofluorescence Analysis

The sections were dewaxed, antigen‐retrieved, and permeabilized before being subjected to overnight incubation with primary antibodies. The primary antibodies used for staining included the following: Sema3E (AF3239, R&D Systems, USA), Plexin D1 (AF4160, R&D Systems, USA), and α‐SMA (55135‐1‐AP, Proteintech, China). The following day, the sections were treated with Alexa 594‐conjugated anti‐rabbit antibody (Invitrogen, CA, USA) and Alexa 488‐conjugated anti‐goat antibody (Servicebio Technology Co. Ltd, China) for 1 h. Nuclei were counterstained with 4′,6‐Diamidino‐2‐phenylindole dihydrochloride (DAPI). Imaging was performed using a fluorescence microscope (Olympus, Tokyo, Japan) at 400× magnification.

### Immunoprecipitation Assay (IP)

Briefly, cells were lysed using NP40, and the supernatant was mixed with 15 µL of blank protein magnetic beads for 2 h in 4 °C, magnetic beads were obtained from MedChemExpress (catalog HY‐K0202). Subsequently, 6 µg of a specific antibody was added to the bead mixture. The antibodies used for the assay were ErbB2 (Proteintech, 18299‐1‐AP), DYKDDDDK tag Polyclonal antibody (20543‐1‐AP, Proteintech, China) and IgG control Polyclonal antibody (30000‐0‐AP, Proteintech, China). The mixture was incubated at 4 °C overnight. Finally, the bound protein was eluted from the magnetic beads and further detected by western blot.

### Western Blot Analysis

Cellular and tissue proteins were extracted using RIPA buffer (Servicebio, China), followed by subjecting sample protein lysates to polyacrylamide gel electrophoresis. Subsequently, proteins were transferred onto polyvinylidene fluoride (PVDF) membranes, which were then blocked with 10% nonfat dried milk (NFDM) freshly prepared in TBS for 1 h. Membranes were incubated with primary antibodies in TTBS/1% NFDM overnight at 4 °C. Following this, the blots were probed with enzyme‐linked secondary antibodies in TTBS/1% NFDM for 1 h at room temperature. Protein signals were detected using ECL Chemiluminescent Substrate (Biosharp, China).

### Enzyme‐Linked Immunosorbent Assay (ELISA)

Sema3E levels were measured in the plasma of healthy controls and IPF patients, as well as in the supernatant of primary human lung fibroblasts, using an ELISA kit (SEL920Hu, Cloud Clone, China). In mice, Sema3E was detected in plasma using a corresponding ELISA kit (SEL920Mu, Cloud Clone, China). Following the manufacturer's instructions, whole blood samples from humans and mice were centrifuged at 800 × g for 10 min, and the plasma supernatant was collected for analysis. The plates were scanned at 450 nm using an enzyme labeling instrument (ELx800, Bio Tek Instruments, Inc., Winooski, VT).

### Cell Migration Assay

Cell migration assays were conducted utilizing Transwell membranes (Corning, MA, USA) with a pore size of 8.0 µm. Cells were suspended in a medium with 1% serum and placed in the upper chamber, whereas the lower chamber contained complete medium with 10% FBS. The treatments and stimuli were applied for 48 h, the cells that had traversed the membrane filter were fixed using 4% paraformaldehyde for 15 min. Subsequently, they were stained with a 20% crystal violet solution for 30 min (C0121, Beyotime Biotechnology, China).

### Cell Proliferation Assay

Cell proliferation was assessed using the EdU proliferation assay (Ribobio, China) as the manufacturer's instructions. In short, cells are seeded into 96‐well plates and cultured, and when cells reached ≈60–70% confluence, the indicated treatments and stimuli were applied for 48 h. The cells are then incubated with Edu solution for 2 h, fixed, and stained with Apollo for half an h. This is followed by staining with Hoechst 33342 to visualize the nuclei. Finally, the cells are examined under a fluorescence microscope at 200× magnification.

### Animal Model

Male C57BL/6J mice (6‐8 weeks) were purchased from Beijing Vital River (Certificate Number: SCXK(E)2022‐0039, China), and housed in a specific pathogen‐free animal center at the Tongji hospital. To induce the pulmonary fibrosis model in mice, the mice were anesthetized with 1% pentobarbital sodium (40 mg kg^−1^) and then intratracheally administered 2 mg kg^−1^ BLM (MedChemExpress, China) in 50 µL of sterile saline with High‐pressure airway atomization drug delivery device (Bio Jane, Cat. No. BJ‐PW‐M, China). Finally, the mice were sacrificed on day 21 after the BLM challenge to analyze pulmonary fibrosis. AAV 9 expressing ShSema3E and control AAV 9 expressing irrelevant sequences for the control were purchased from the OBIO Technology (Shanghai, China). The AAV virus was quantified and administered in genome copies (GC). Specifically, C57BL/6 mice (8‐week‐old, male) were anesthetized and intratracheally administered with AAV9‐ShSema3E (2 × 10^11^ GC per mouse) and AAV9‐NC (2 × 10^11^ GC per mouse). Modeling of pulmonary fibrosis in mice started 14 days after intratracheally administered AAV9‐ShSema3E and AAV9‐NC and the mice were sacrificed on day 21 after the BLM challenge.

Sema3E flox/flox mice on a C57BL/6 background were generated by GemPharmatech (Jiangsu, China) using CRISPR‐Cas9, with loxP sites flanking exons 6–9 (Figure S6A, Supporting Information). Coll1a2‐iCre transgenic mice were obtained from Sayer Biologicals (Jiangsu, China) and crossed with Sema3E flox/flox mice to generate Coll1a2‐iCre^+^ Sema3E flox/flox mice (referred to as Sema3E‐CKO). Fibroblast‐specific knockout of Sema3E was validated by Western blot and immunofluorescence analysis of lung tissue. Littermate mice lacking Cre (Col1a2‐iCre^−^ Sema3E flox/flox, referred to as Sema3E‐C) served as controls. Male Sema3E‐CKO and Sema3E‐C mice, aged 6–8 weeks, were used for bleomycin airway injection to investigate the role of fibroblast‐specific Sema3E knockout in pulmonary fibrosis. Additionally, starting 7 days after airway administration of BLM, experimental mice received intraperitoneal injections of Hexa‐D‐arginine (Furin inhibitor, 1.5 mg kg^−1^) or an equivalent‐volume PBS every other day for the following 14 days.

### Pathological Staining and Histopathologic Assessment

Following the euthanasia of the mice 21 days post‐administration of either BLM or saline, lung tissues were excised and fixed in 4% paraformaldehyde. Subsequently, the fixed lung tissues were embedded in paraffin and sectioned. Sections were stained with hematoxylin and eosin (H&E) staining, Masson's trichrome staining, and Sirius red staining. Fibrosis was scored using the Ashcroft scale, which ranged from 0 to 8. Three randomly selected areas from each mouse lung were assessed at 200× magnification, and the severity of fibrotic alteration was evaluated as the mean of the scores obtained from these microscopic fields. The scoring process was performed independently by two observers in a blinded manner.

### Hydroxyproline Assay

Lung collagen deposition was quantified by measuring the hydroxyproline content in lung homogenates using a hydroxyproline assay kit (Jiancheng, Nanjing, China) according to the manufacturer's instructions. Briefly, 30 mg of lung tissue was accurately weighed, and 1 mL of hydrolysate was added. The mixture was heated in boiling water for 20 min, then cooled. After cooling, the pH was adjusted to 6.0–6.8, and the final volume was adjusted to 7 mL with double‐distilled water. A 2 mL aliquot of the sample was then mixed with 20 mg of activated carbon, followed by centrifugation at 3500 RPM for 10 min. The supernatant (1 mL) was collected as the test sample. The test reagent from the kit was added, and the sample was incubated at 60 °C for 15 min. Following incubation, the sample was centrifuged again at 3500 RPM for 10 min. The resulting supernatant was transferred to a 96‐well plate, and absorbance was measured at 550 nm. Hydroxyproline concentration was determined by comparison to a standard sample and normalized to the initial lung tissue weight. Hydroxyproline content is expressed as µg of hydroxyproline per mg of lung tissue.

### Statistical Analyses

Analyses of the aforementioned measurements obtained from at least three independent experiments were conducted using GraphPad Prism 9.0.0. The statistical outcomes are presented as means ± standard errors of the mean (SEMs). Unpaired t‐tests were utilized to compare between two groups, whereas one‐way ANOVA was used for statistical analysis involving multiple groups. Correlations were analyzed using Pearson's correlation. Statistical significance was defined as *p* < 0.05, with significance levels further categorized as follows: *p* < 0.05, *p* < 0.01, and *p* < 0.001.

## Conflict of Interest

The authors declare no conflict of interest.

## Author Contributions

J.X. and A.G. designed the study. L.S. provided theoretical guidance. Z.D. performed the experiments, analyzed the data, generated the figures and tables, and wrote the manuscript. R.Y. performed part of the experiments. Y.Z., Z.D., R.Y., S.C., J.Z., H.F., Q.H., J.W., and Y.G. collected the clinical lung tissue specimens and plasma. J.C. and J.X. supervised the program and edited the manuscript. All authors reviewed the final manuscript and approved the submission.

## Supporting information



Supporting Information

## Data Availability

The data that support the findings of this study are available from the corresponding author upon reasonable request.

## References

[1] L. Richeldi , H. R. Collard , M. G. J. T. L. Jones , Lancet Respir. Med. 2017, 389, 1941.

[2] V. Somogyi , N. Chaudhuri , S. E. Torrisi , N. Kahn , V. Müller , M. Kreuter , Eur. Respir. Rev. 2019, 28, 190021. 31484664 10.1183/16000617.0021-2019 PMC9488691

[3] E. El Agha , A. Moiseenko , V. Kheirollahi , S. De Langhe , S. Crnkovic , G. Kwapiszewska , M. Szibor , D. Kosanovic , F. Schwind , R. T. Schermuly , I. Henneke , B. MacKenzie , J. Quantius , S. Herold , A. Ntokou , K. Ahlbrecht , T. Braun , R. E. Morty , A. Günther , W. Seeger , S. Bellusci , Cell Stem Cell 2017, 20, 261. 27867035 10.1016/j.stem.2016.10.004 PMC5291816

[4] C. Sack , G. Raghu , Eur. Respir. J. 2019, 53, 1801699. 30487201 10.1183/13993003.01699-2018

[5] M. Selman , T. E. King , A. Pardo , Ann. Intern. Med. 2001, 134, 136. 11177318 10.7326/0003-4819-134-2-200101160-00015

[6] A. L. Kolodkin , D. J. Matthes , C. S. Goodman , Cell 1993, 75, 1389. 8269517 10.1016/0092-8674(93)90625-z

[7] F. De Angelis Rigotti , L. Wiedmann , M. O. Hubert , M. Vacca , S. S. Hasan , I. Moll , S. Carvajal , W. Jiménez , M. Starostecka , A. T. Billeter , B. Müller‐Stich , G. Wolff , B. Ekim‐Üstünel , S. Herzig , C. Fandos‐Ramo , R. Krätzner , M. Reich , V. Keitel‐Anselmino , M. Heikenwälder , C. Mogler , A. Fischer , J. Rodriguez‐Vita , Hepatology (Baltimore, Md.) 2023, 78, 1092. 10.1097/HEP.0000000000000407 37055018

[8] T. Carvalheiro , A. J. Affandi , B. Malvar‐Fernández , I. Dullemond , M. Cossu , A. Ottria , J. S. Mertens , B. Giovannone , F. Bonte‐Mineur , M. R. Kok , W. Marut , K. A. Reedquist , T. R. Radstake , S. García , Arthritis Rheumatol. (Hoboken, N.J.) 2019, 71, 1711. 10.1002/art.40915 PMC6790618 31012544

[9] Y. Sang , K. Tsuji , K. Fukushima , K. Takahashi , S. Kitamura , J. Wada , Renal Physiology 2021, 321, F740. 34747196 10.1152/ajprenal.00234.2021

[10] R. A. Reilkoff , H. Peng , L. A. Murray , X. Peng , T. Russell , R. Montgomery , C. Feghali‐Bostwick , A. Shaw , R. J. Homer , M. Gulati , A. Mathur , J. A. Elias , E. L. Herzog , Am J. Respir. Crit. Care Med. 2013, 187, 180. 23220917 10.1164/rccm.201206-1109OC PMC3570653

[11] B. Jiao , S. Liu , X. Tan , P. Lu , D. Wang , H. Xu , Biomedicine & pharmacotherapy = Biomedecine & Pharmacotherapie 2021, 137, 111329. 33545660 10.1016/j.biopha.2021.111329

[12] S. M. Kanth , S. Gairhe , P. Torabi‐Parizi , Frontiers in Immunology 2021, 12, 672441. 34012455 10.3389/fimmu.2021.672441 PMC8126651

[13] A. Casazza , V. Finisguerra , L. Capparuccia , A. Camperi , J. M. Swiercz , S. Rizzolio , C. Rolny , C. Christensen , A. Bertotti , I. Sarotto , M. Risio , L. Trusolino , J. Weitz , M. Schneider , M. Mazzone , P. M. Comoglio , L. Tamagnone , J. Clin. Invest. 2010, 120, 2684. 20664171 10.1172/JCI42118 PMC2912191

[14] J. Luchino , M. Hocine , M. C. Amoureux , B. Gibert , A. Bernet , A. Royet , I. Treilleux , P. Lécine , J. P. Borg , P. Mehlen , S. Chauvet , F. Mann , Cancer Cell 2013, 24, 673. 24139859 10.1016/j.ccr.2013.09.010

[15] K. Hagihara , N. Haraguchi , J. Nishimura , A. Yasueda , S. Fujino , T. Ogino , H. Takahashi , N. Miyoshi , M. Uemura , C. Matsuda , T. Mizushima , H. Yamamoto , M. Mori , Y. Doki , H. Eguchi , Annals of Surgical Oncology 2022, 29, 7435. 35917012 10.1245/s10434-022-11945-y

[16] C. Mazzotta , E. Romano , C. Bruni , M. Manetti , G. Lepri , S. Bellando‐Randone , J. Blagojevic , L. Ibba‐Manneschi , M. Matucci‐Cerinic , S. Guiducci , Arthritis Res. Ther. 2015, 17, 221. 26292963 10.1186/s13075-015-0749-4 PMC4546224

[17] T. Yagai , A. Miyajima , M. Tanaka , Am. J. Pathol. 2014, 184, 2250. 24930441 10.1016/j.ajpath.2014.04.018

[18] H. Movassagh , F. Khadem , A. S. Gounni , Am. J. Respir. Cell Mol. Biol. 2018, 58, 21. 28817310 10.1165/rcmb.2017-0171TR

[19] R. H. Adams , M. Lohrum , A. Klostermann , H. Betz , A. W. Püschel , EMBO J. 1997, 16, 6077. 9321387 10.1093/emboj/16.20.6077 PMC1326291

[20] C. Christensen , N. Ambartsumian , G. Gilestro , B. Thomsen , P. Comoglio , L. Tamagnone , P. Guldberg , E. Lukanidin , Cancer Res. 2005, 65, 6167. 16024618 10.1158/0008-5472.CAN-04-4309

[21] A. Casazza , B. Kigel , F. Maione , L. Capparuccia , O. Kessler , E. Giraudo , M. Mazzone , G. Neufeld , L. Tamagnone , EMBO Mol. Med. 2012, 4, 234. 22247010 10.1002/emmm.201100205 PMC3376853

[22] H. Movassagh , L. Shan , J. Chakir , J. F. McConville , A. J. Halayko , L. Koussih , A. S. Gounni , The Journal of Allergy and Clinical Immunology 2017, 140, 1176. 28506853 10.1016/j.jaci.2017.04.031

[23] H. Movassagh , L. Shan , J. S. Duke‐Cohan , A. J. Halayko , J. E. Uzonna , A. S. Gounni , Am. J. Pathol. 2017, 187, 1566. 28634005 10.1016/j.ajpath.2017.03.008

[24] M. Matloubi , L. Koussih , L. Shan , C. Lukawy , A. S. Gounni , Pharmacol. Ther. 2023, 242, 108351. 36706796 10.1016/j.pharmthera.2023.108351

[25] P. Bradding , M. Richardson , T. S. C. Hinks , P. H. Howarth , D. F. Choy , J. R. Arron , S. E. Wenzel , S. Siddiqui , The Journal of Allergy and Clinical Immunology 2020, 146, 208. 32450087 10.1016/j.jaci.2020.05.013 PMC7243787

[26] Z. He , A. M. Khatib , J. W. M. Creemers , Oncogene 2022, 41, 1252. 34997216 10.1038/s41388-021-02175-9

[27] P. Jaaks , M. Bernasconi , Int. J. Cancer 2017, 141, 654. 28369813 10.1002/ijc.30714

[28] J. Yin , H. M. Chang , Y. Yi , Y. Yao , P. C. K. Leung , Cells 2020, 9. 10.3390/cells9010185 PMC7016865 31936902

[29] Y. Wang , L. Zhang , T. Huang , G. R. Wu , Q. Zhou , F. X. Wang , L. M. Chen , F. Sun , Y. Lv , F. Xiong , S. Zhang , Q. Yu , P. Yang , W. Gu , Y. Xu , J. Zhao , H. Zhang , W. Xiong , C. Y. Wang , Eur. Respir. J. 2022, 60, 2003697. 35086828 10.1183/13993003.03697-2020 PMC9520034

[30] L. Wollin , E. Wex , A. Pautsch , G. Schnapp , K. E. Hostettler , S. Stowasser , M. Kolb , Eur. Respir. J. 2015, 45, 1434. 25745043 10.1183/09031936.00174914 PMC4416110

[31] C. L. Arteaga , J. A. Engelman , Cancer Cell 2014, 25, 282. 24651011 10.1016/j.ccr.2014.02.025 PMC4018830

[32] X. Liu , Y. Geng , J. Liang , A. L. Coelho , C. Yao , N. Deng , Y. Wang , K. Dai , G. Huang , T. Xie , N. Liu , S. C. Rowan , F. Taghavifar , V. Kulur , Z. Liu , B. R. Stripp , C. M. Hogaboam , D. Jiang , P. W. Noble , J. Exp. Med. 2022, 219, e20220126. 35980387 10.1084/jem.20220126 PMC9391950

[33] J. Wang , K. Hu , X. Cai , B. Yang , Q. He , J. Wang , Q. Weng , Acta Pharmaceutica Sinica. B 2022, 12, 18. 35127370 10.1016/j.apsb.2021.07.023 PMC8799876

[34] H. Matsuoka , T. Arai , M. Mori , S. Goya , H. Kida , H. Morishita , H. Fujiwara , I. Tachibana , T. Osaki , S. Hayashi , Lung Cellular and Molecular Physiology 2002, 283, L103. 12060566 10.1152/ajplung.00187.2001

[35] P. R. Gajjala , R. K. Kasam , D. Soundararajan , D. Sinner , S. K. Huang , A. G. Jegga , S. K. Madala , JCI insight 2021, 6, e152503. 34520400 10.1172/jci.insight.152503 PMC8564901

