# Supplementary material for: Semaphorin 3E–Plexin D1 Axis Drives Lung Fibrosis through ErbB2‐Mediated Fibroblast Activation
Source: Adv Sci (Weinh). 2025 Mar 20;12(18):2415007. doi: 10.1002/advs.202415007 (PMC12079525; doi:10.1002/advs.202415007)
Supplement: Supplementary file 1 — Supporting Information [file ADVS-12-2415007-s001.docx]

**Supplementary figures**


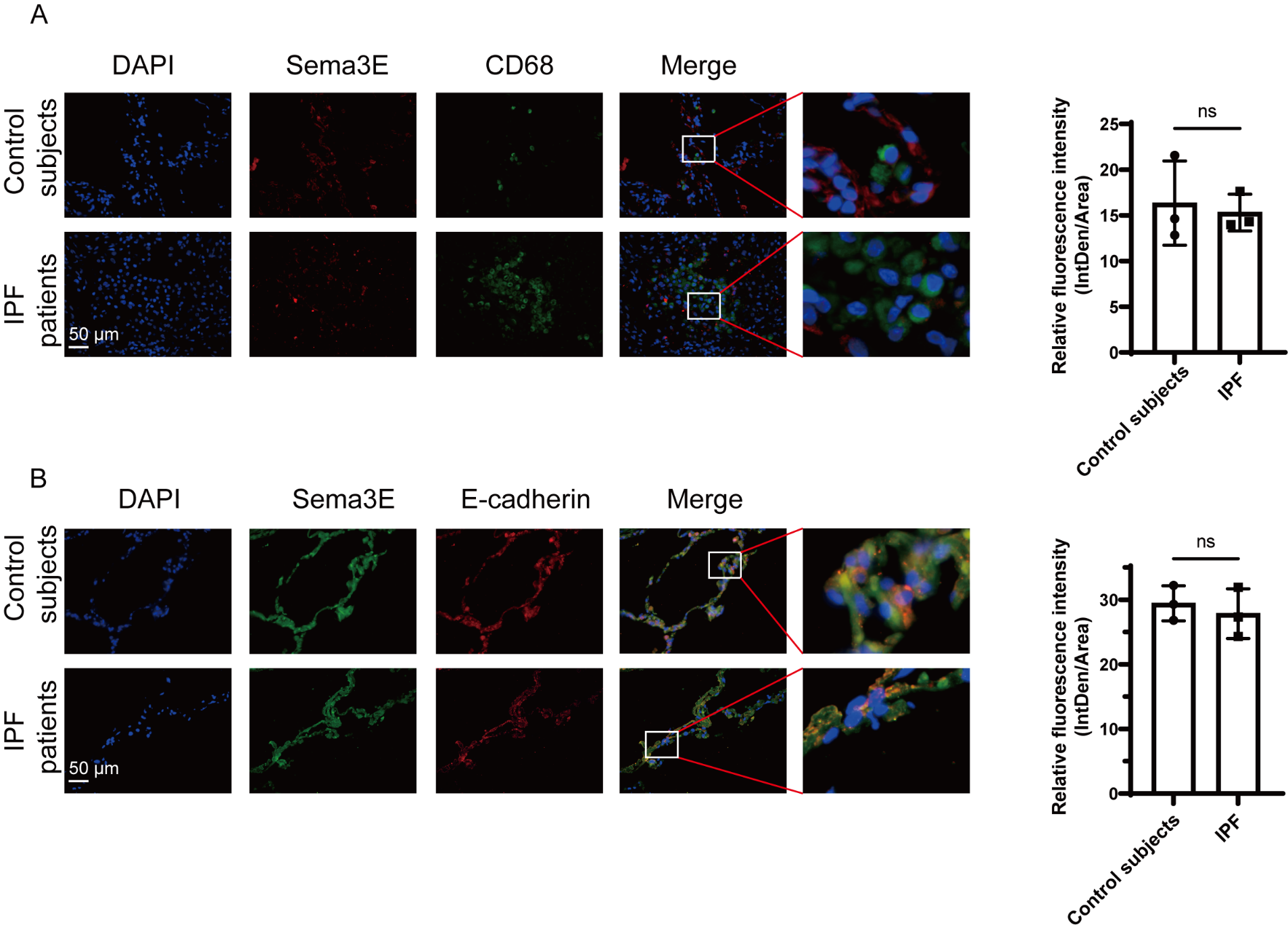


Figure S1. Analysis of Sema3E expression in macrophages and alveolar Epithelial cells in patients with IPF. (A) Co-immunostaining of Sema3E and CD68 (macrophage maker) in lung sections from patients with IPF (n=3) and control subjects(n=3). (B) Co-immunostaining of Sema3E and E-cadherin (alveolar epithelial cell) in lung sections from patients with IPF(n=3) and control subjects (n=3). Nuclei were stained blue with DAPI, and images were captured under original magnification ×400. Statistical analyses were performed using unpaired t-tests. Data are represented as the mean ± SEM. ns (no significant difference) indicates that the results do not show a statistically significant difference between compared groups (typically p > 0.05).


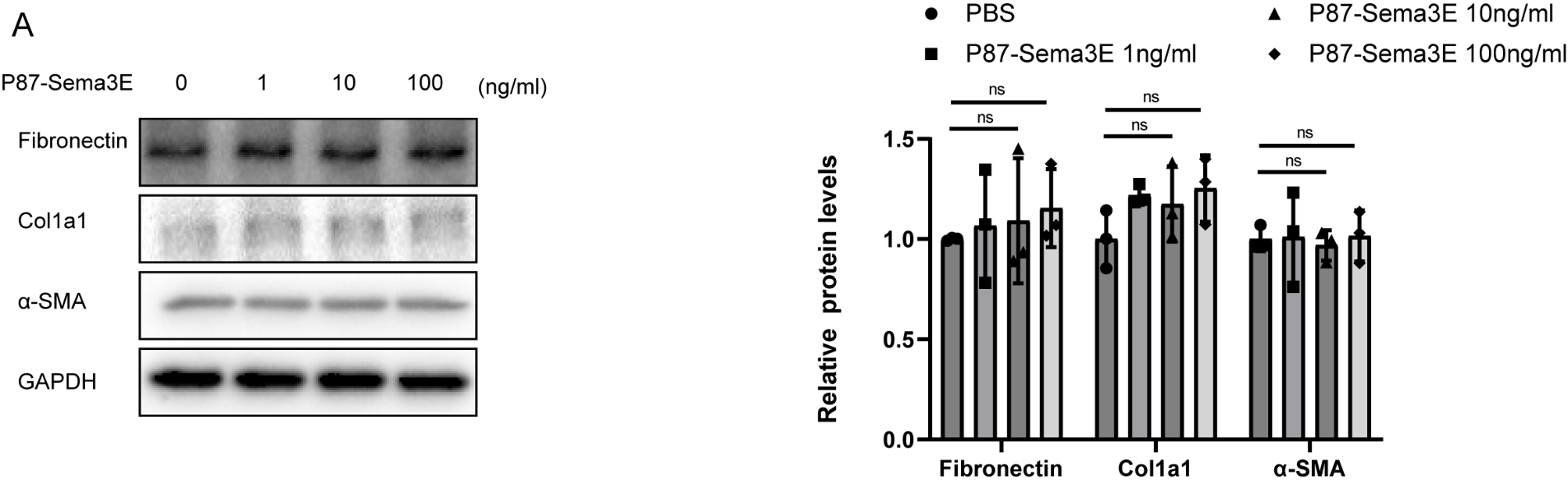


Figure S2. Effect of p87-Sema3E on fibroblast activation. (A) Western blot analysis of the levels of Fibronectin, Col1a1, and α-SMA in primary human lung fibroblasts after stimulation with different concentrations of P87-Sema3E for 48 hours. Statistical analyses were performed using one-way ANOVA tests. Data are represented as the mean ± SEM. ns (no significant difference) indicates that the results do not show a statistically significant difference between compared groups (typically p > 0.05).


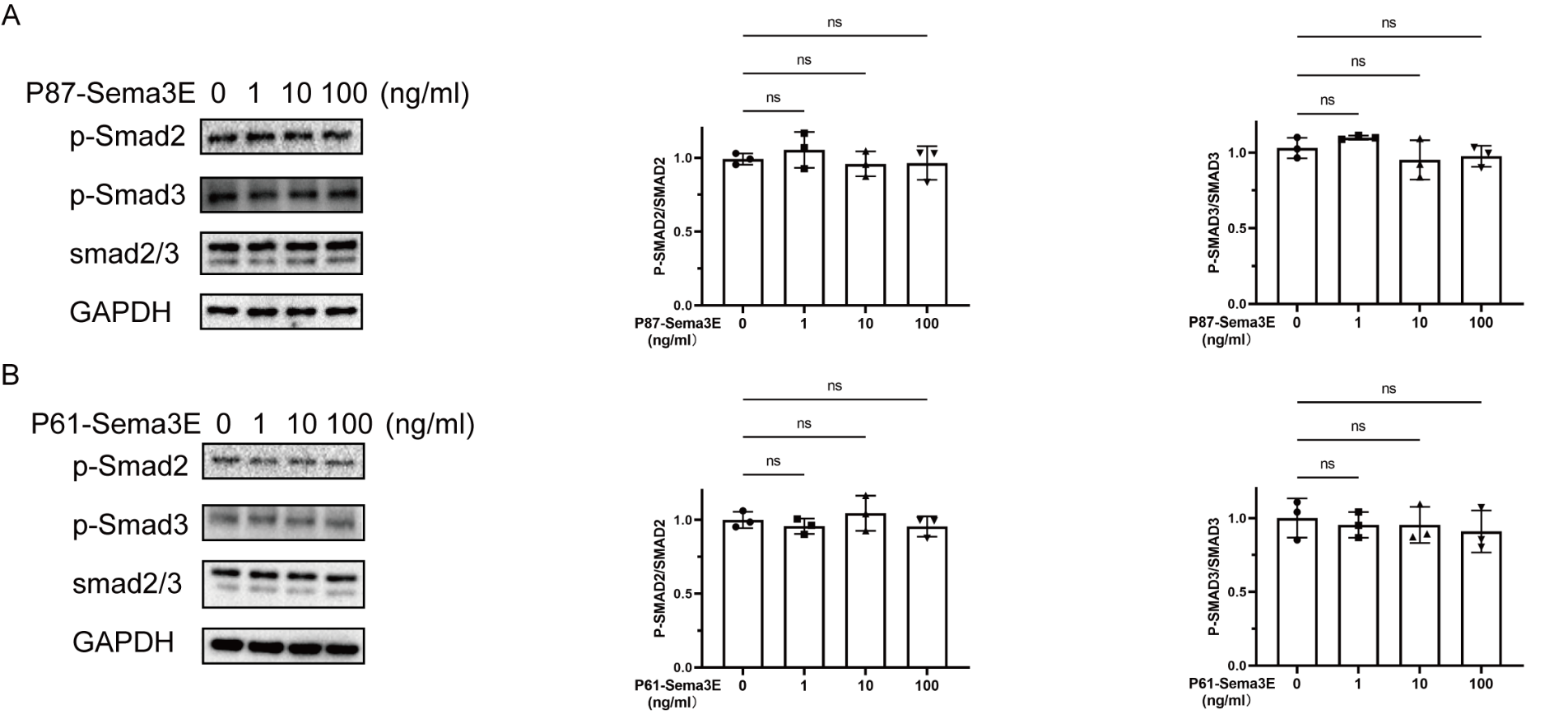


Figure S3. Effects of Sema3E on the TGF-β Pathway. (A) Western blot analysis of the levels of P-Smad2, P-Smad3 and Smad2/3 in primary human lung fibroblasts after stimulation with different concentrations of P87-Sema3E for 2 hours. (B) Western blot analysis of the levels of P-Smad2, P-Smad3 and Smad2/3 in primary human lung fibroblasts after stimulation with different concentrations of P61-Sema3E for 2 hours. Data are represented as the mean ± SEM. Statistical analyses were performed using one-way ANOVA tests of three independent experiments. ns (no significant difference) indicates that the results do not show a statistically significant difference between compared groups (typically p > 0.05).


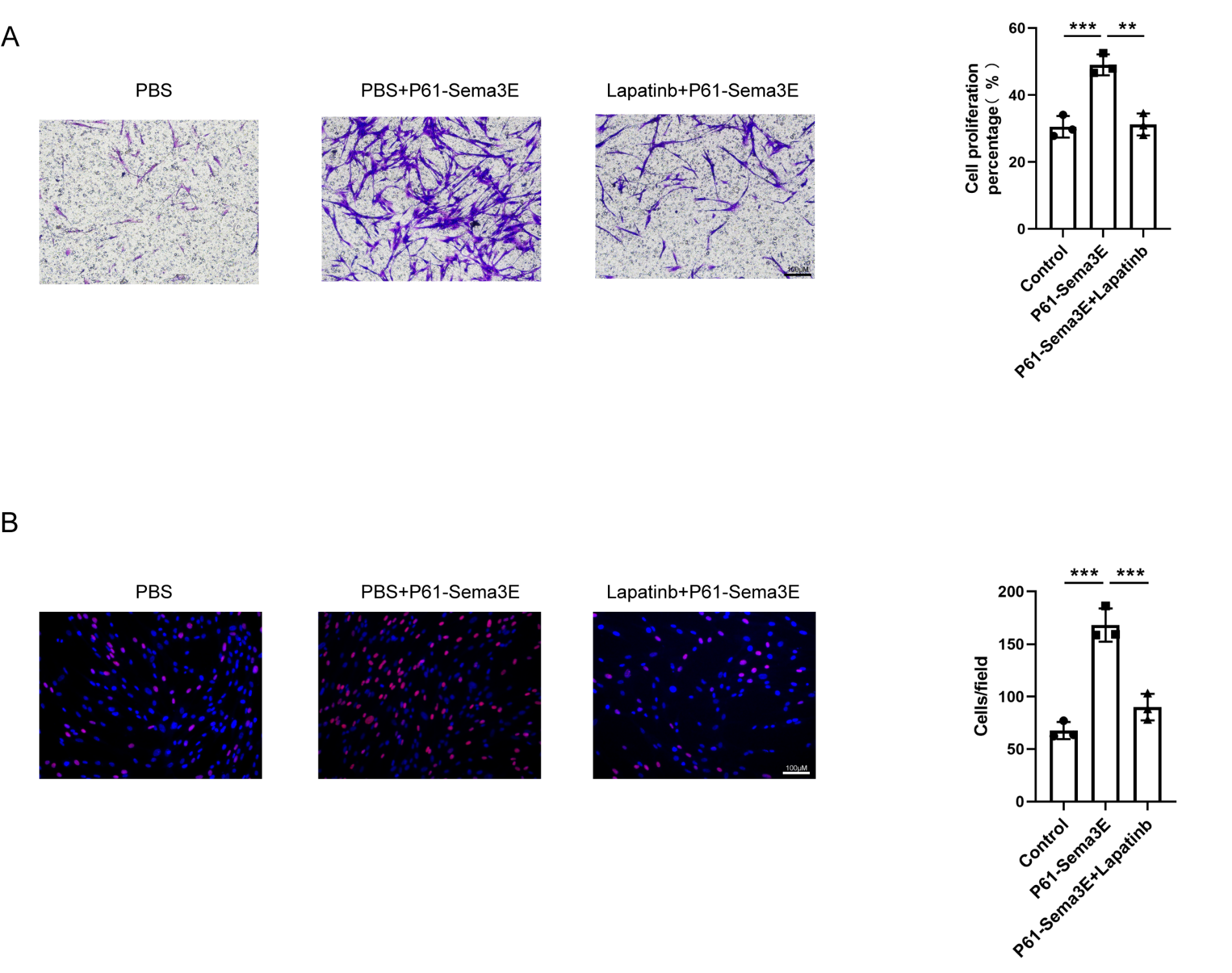


Figure S4. Inhibitory effect of ErbB2 inhibitor Lapatinib on P61-Sema3E promoting fibrosis. (A-B) Representative results for EdU staining and Transwell assay in primary human lung fibroblasts treated with the ErbB2 inhibitor Lapatinib or PBS following P61-Sema3E stimulation. Nuclei were stained blue with DAPI, and images were captured under original magnification ×200. Data are represented as the mean ± SEM. Statistical analyses were performed using one-way ANOVA tests of three independent experiments. **p < 0.01; ***p < 0.001.


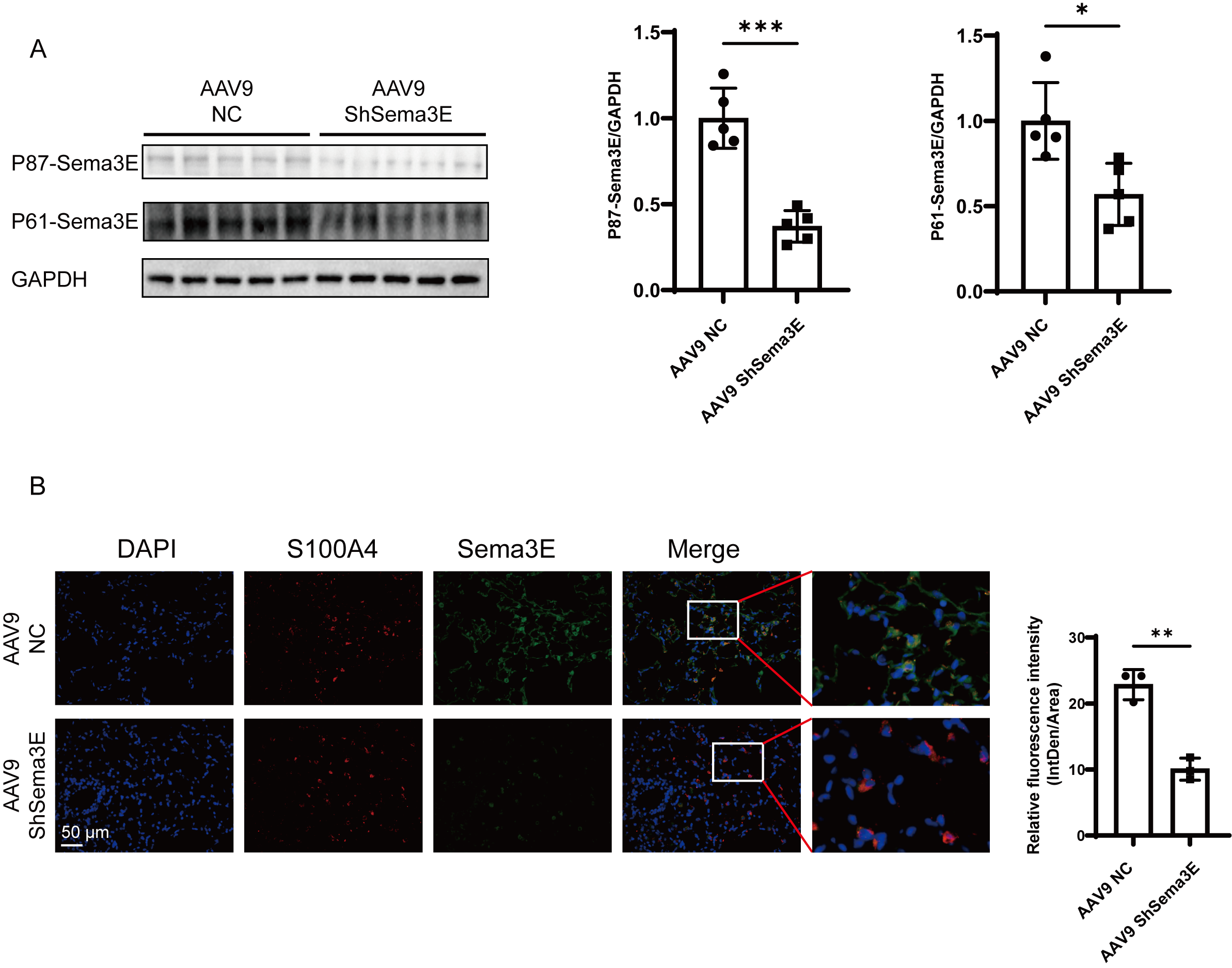


Figure S5. AAV-Mediated knockdown reduces Sema3E expression in mouse lung Tissue. (A) Western blot analysis of P87-Sema3E and P61-Sema3E in lung tissues from AAV9-NC saline mice (n=5) and AAV9-shSema3E saline mice (n=5). (B) Immunofluorescence detection of Sema3E in lung tissues from AAV9-NC saline mice (n=3) and AAV9-shSema3E saline mice (n=3). Nuclei were stained blue with DAPI, and images were captured under original magnification ×400. Data are presented as the mean ± SEM. Statistical analyses were performed using unpaired t-tests. *p < 0.05; **p < 0.01; ***p < 0.001. Abbreviation: AAV, adeno-associated virus.


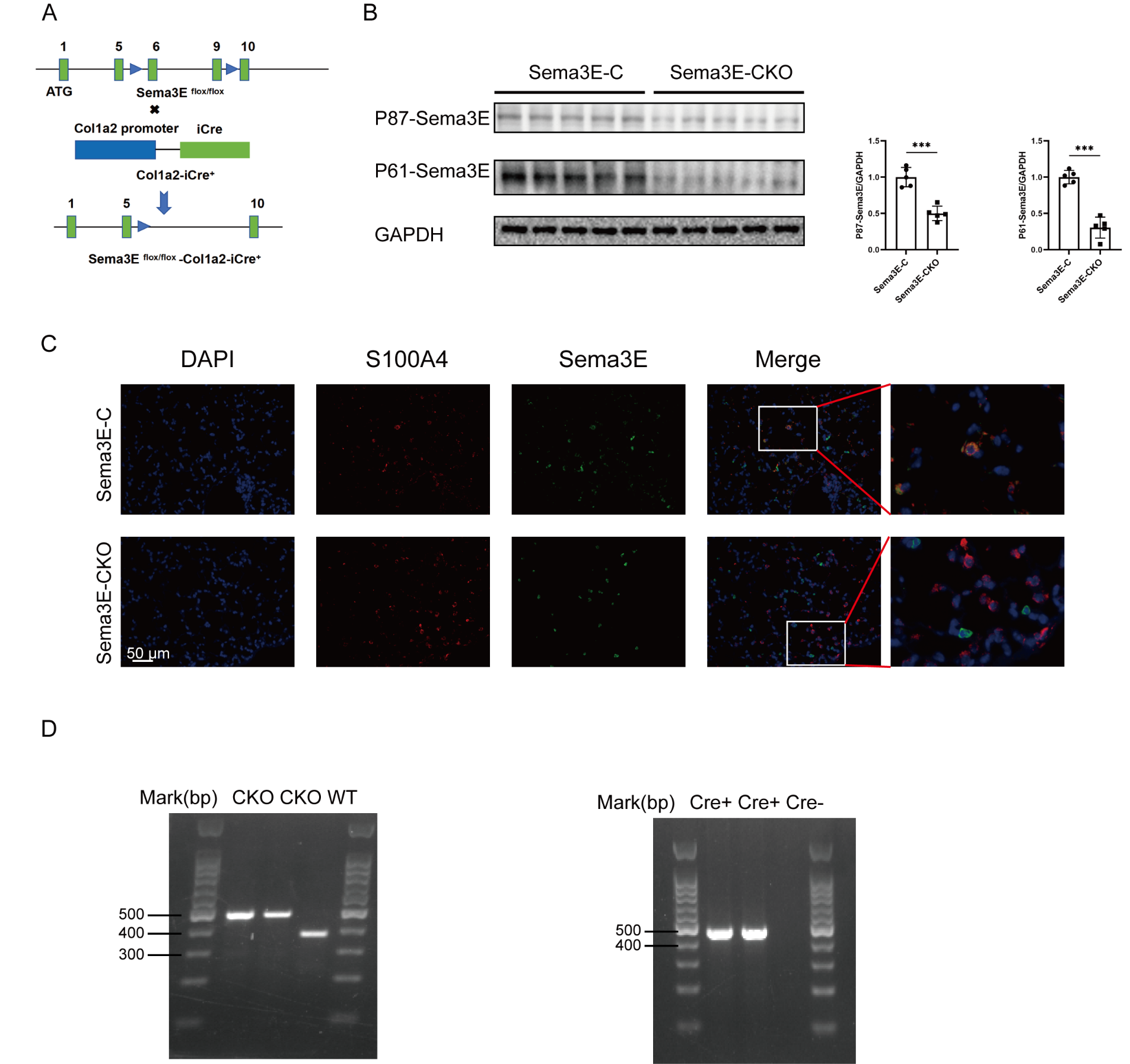


Figure S6. The generation of fibroblast deletion of Sema3E mice. (A)The Sema3E-flox/flox mice in C57BL/6JGpt background were generated using the clustered regularly interspaced short palindromic repeats (CRISPR)–Cas9 system. Two loxP sequences were inserted in the introns flanking exon 6-9 of Sema3E, as described in figure. Coll1a2-iCre transgenic mice crossed with Sema3E flox/flox mice to generate Coll1a2-iCre^+^ Sema3E flox/flox mice. (B) Western blot analysis of the levels of P87-Sema3E and P61-Sema3E in mouse lung tissue. Samples include Sema3E-C mice (n=5), Sema3E-CKO mice (n=5). (C) Representative results for co-immunostaining of S100A4 and Sema3E in lung sections from Sema3E-C (n=3) and Sema3E-CKO mice (n=3). Nuclei were stained blue with DAPI, and images were captured under original magnification ×400. (D) PCR analysis of DNA to determine the presence of the floxed allele (left) and the cre allele (right). Data are represented as the mean ± SEM. Statistical analyses were performed using unpaired t-tests. ***p < 0.001.


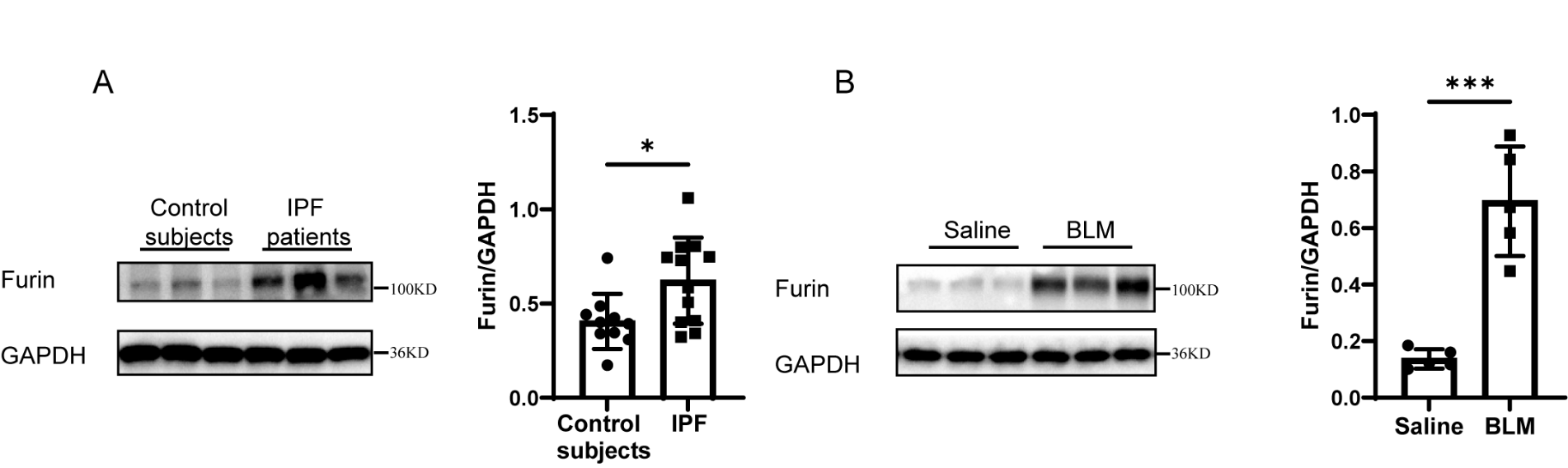


Figure S7. Furin expression was detected in lung tissues of IPF patients and BLM-induced mice. (A)Western blot analysis of the levels of Furin in lung tissue samples from control subjects (n = 10) and IPF patients (n = 12). (B) Western blot analysis of the levels of Furin in lung homogenates from saline-treated (n=5) and BLM-induced (n=5) mouse models. Data are represented as the mean ± SEM. Statistical analyses were performed using unpaired t-tests. *p < 0.05; ***p < 0.001.

**Table S1**

Summary of clinicopathological characteristics of Plasma samples.

|  | Controls | IPF |
| --- | --- | --- |
| Gender(male/female) | 15/9 | 37/26 |
| Age(years) | 52.83±5.75 | 59.78±11.53 |
| FVC% pred | 120.49±17.17 | 81.30±19.37 |
| FVC absolute L | 3.90±0.70 | 2.46±0.64 |
| FEV1% pred | 113.58±13.04 | 79.72±19.42 |
| FEV1 absolute L | 3.06±0.52 | 1.95±0.54 |
| DLCO% pred |  | 55.40±15.84 |
| TLC% pred |  | 72.99±12.53 |

IPF: idiopathic pulmonary fibrosis; % pred: % predicted; FVC: forced vital capacity; FEV1: forced expiratory volume in 1 s; DLCO: diffusing capacity of the lung for carbon monoxide; TLC: total lung capacity.

**Table S2**

Summary of clinicopathological characteristics of lung tissue samples.

|  | Controls | IPF |
| --- | --- | --- |
| Gender(male/female) | 9/1 | 11/1 |
| Age(years) | 53.80±5.75 | 52.83±9.50 |

**Table S3**

The primer sequences utilized are as follows:

| Human  Fibronectin | forward | 5′-ACAACACCGAGGTGACTGAGAC-3′ |
| --- | --- | --- |
|  | reverse | 5′-GGACACAACGATGCTTCCTGAG-3′ |
| Human  COL1A1 | forward | 5′-GATTCCCTGGACCTAAAGGTGC-3′ |
|  | reverse | 5′-AGCCTCTCCATCTTTGCCAGCA-3′ |
| Human  ACTA2 | forward | 5′-TGCTGACAGAGGCACCACTGAA-3 |
|  | reverse | 5′-CAGTTGTACGTCCAGAGGCATAG-3′ |
| Human  GAPDH | forward | 5′-GTCTCCTCTGACTTCAACAGCG-3′ |
|  | reverse | 5′-ACCACCCTGTTGCTGTAGCCAA-3′ |
| Mouse  Fn-1 | forward | 5′-CCCTATCTCTGATACCGTTGTCC-3′ |
|  | reverse | 5′-TGCCGCAACTACTGTGATTCGG-3′ |
| Mouse  Col1a1 | forward | 5′-CCTCAGGGTATTGCTGGACAAC-3′ |
|  | reverse | 5′-CAGAAGGACCTTGTTTGCCAGG-3′ |
| Mouse  Acta2 | forward | 5′-TGTGACAGAGGCACCACTGAA-3′ |
|  | reverse | 5′-CAGTTGTACGTCCAGAGGCATAG-3′ |
| Mouse  Gapdh | forward | 5′-CATCACTGCCACCCAGAAGACTG-3′ |
|  | reverse | 5′-ATGCCAGTGAGCTTCCCGTTCAG-3′ |
